# Machine Learning-Guided Protein Engineering

**DOI:** 10.1021/acscatal.3c02743

**Published:** 2023-10-13

**Authors:** Petr Kouba, Pavel Kohout, Faraneh Haddadi, Anton Bushuiev, Raman Samusevich, Jiri Sedlar, Jiri Damborsky, Tomas Pluskal, Josef Sivic, Stanislav Mazurenko

**Affiliations:** 1 Loschmidt Laboratories, Department of Experimental Biology and RECETOX, Faculty of Science, Masaryk University, Kamenice 5, 625 00 Brno, Czech Republic; 2 International Clinical Research Center, St. Anne’s University Hospital Brno, Pekarska 53, 656 91 Brno, Czech Republic; 3 Czech Institute of Informatics, Robotics and Cybernetics, Czech Technical University in Prague, Jugoslavskych partyzanu 1580/3, 160 00 Prague 6, Czech Republic; 4 Faculty of Electrical Engineering, Czech Technical University in Prague, Technicka 2, 166 27 Prague 6, Czech Republic; 5 Institute of Organic Chemistry and Biochemistry of the Czech Academy of Sciences, Flemingovo nám. 2, 160 00 Prague 6, Czech Republic

**Keywords:** activity, artificial intelligence, biocatalysis, deep learning, protein design

## Abstract

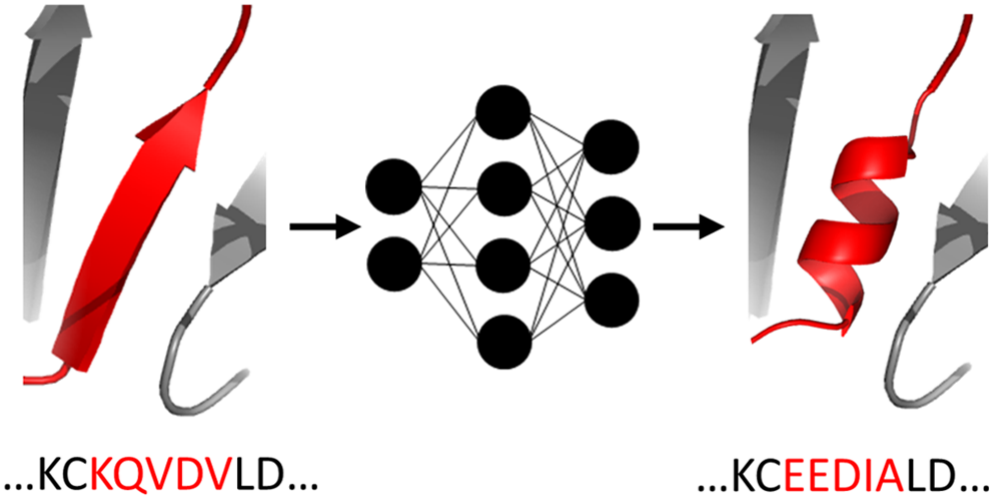

Recent progress in engineering highly promising biocatalysts has increasingly involved machine learning methods. These methods leverage existing experimental and simulation data to aid in the discovery and annotation of promising enzymes, as well as in suggesting beneficial mutations for improving known targets. The field of machine learning for protein engineering is gathering steam, driven by recent success stories and notable progress in other areas. It already encompasses ambitious tasks such as understanding and predicting protein structure and function, catalytic efficiency, enantioselectivity, protein dynamics, stability, solubility, aggregation, and more. Nonetheless, the field is still evolving, with many challenges to overcome and questions to address. In this Perspective, we provide an overview of ongoing trends in this domain, highlight recent case studies, and examine the current limitations of machine learning-based methods. We emphasize the crucial importance of thorough experimental validation of emerging models before their use for rational protein design. We present our opinions on the fundamental problems and outline the potential directions for future research.

## 1 Introduction

Biocatalysis is a promising field that offers diverse possibilities for creating sustainable and environmentally friendly solutions in various industries. Its potential stems from its ability to mimic and harness the power of nature using cells and enzymes that have evolved over millions of years to perform specific chemical reactions with high efficiency. This makes it possible to transform chemical compounds selectively and efficiently, providing an alternative to traditional chemical catalysis, which often requires harsh conditions and toxic chemicals.^1^ Biocatalysts could therefore be valuable in the production of fine chemicals, pharmaceuticals, and food ingredients as well as in the development of sustainable processes for the production of energy and materials.^2^ In addition, biocatalysis is an exciting area of research and development with great promise for the future because of the potential to unlock new solutions for diverse challenges by providing green alternatives to traditional chemical processes, new energy sources, and tools for improving the overall efficiency of industrial processes or biological removal of recalcitrant waste.^3−5^ It is also a highly interdisciplinary research area that makes heavy use of advanced experimental techniques and computational methods.^6^

Many research fields are undergoing a gradual transition from near-exclusive reliance on experimental work to hybrid approaches that incorporate computational simulations and data-driven methods.^7−9^ In the past, researchers would accumulate observations from individual experiments and use the resulting data to formulate fundamental rules. They then created simulations based on these rules to better understand the system under investigation. As computational power has increased, researchers have been able to shift toward data-driven methods that rely on **machine learning** (ML, see the glossary in Table 1 for terms in bold) algorithms to deduce rules directly from data.^10,11^ This transition has made it possible to efficiently and comprehensively analyze large and complex data sets that are often generated by high-throughput technologies. Particularly, very powerful **deep learning** algorithms are finding a wide range of applications in life sciences and will be discussed in great detail in this Perspective. While experimental science and computational simulations still play essential roles, the trend toward data-driven methods will likely continue as technology and data collection methods evolve further.^7^

**Table 1:** Glossary of Terms Used Frequently in the Context of Machine Learning for Enzyme Engineering^a^

accuracy	metric primarily used for classification tasks, measuring the ratio of correct predictions to all predictions produced by a machine learning model
active learning	a type of machine learning in which the learning algorithm queries a person for providing labels for particular data points during training iteratively. the first iteration usually starts with many unlabeled and few labeled data points, *e.g.*, protein sequences. after training on this data set, the algorithm proposes a next set of data points for labeling to the experimenter, *e.g.*, more sequences to be characterized in the lab. their labels are then provided to the algorithm for the next iteration, and the cycle repeats several times
artificial intelligence (AI)	artificial intelligence as defined by McCarthy is “the science and engineering of making intelligent machines, especially intelligent computer programs”
continual learning	a concept in which a model can train on new data while maintaining the abilities acquired from earlier training on old data
cross-validation (*K*-fold)	an approach to evaluating the performance of a model whereby a data set is split into *K* parts, the model is retrained *K* times on all but one part, and the performance is evaluated on the excluded part. this way each data point is used once for validation, and *K* different evaluations are produced to provide a distribution of the values
deep learning (DL)	a branch of machine learning that uses multiple-layer neural network architectures. deep networks generally include many more parameters (sometimes, billions) and hyperparameters than traditional machine learning models. this gives deep neural networks tremendous expressive power and design flexibility, which has made them a major driver of modern technology with applications ranging from on-the-fly text generation to protein structure prediction
diffusion	deep learning paradigm based on denoising diffusion probabilistic modeling. diffusion models learn to generate novel objects, *e.g.*, images or proteins, by reconstructing artificially corrupted training examples
embedding	representation of high-dimensional data, *e.g.*, text, images, or proteins, in a lower-dimensional vector space while preserving important information
end-to-end learning	a type of ML that requires minimal to no data transformation (*e.g.*, just one-hot encoding of the input) to train a predictor. This is often the case in deep learning, when abundant data are available to establish direct input-to-output correspondence, in contrast to classical ML approaches using small data sets, which typically require feature and label engineering before the data can be used for training
equivariance	an ML model is said to be equivariant with respect to a particular transformation if the order of applying the transformation and the model to an input does not change the outcome. for example, if we pass a rotated input to a model that is equivariant to rotation, the result will be the same as if the model was applied to the original input and the output was then rotated
explainable artificial intelligence (XAI)	AI or ML-based algorithms designed such that humans can understand the reasons for their predictions. its core principles are transparency, interpretability, and explainability
findable, accessible, interoperable, reusable (FAIR principles)	principles for the management and stewardship of scientific data to ensure findability, accessibility, interoperability, and reusability
fine-tuning	an approach to transfer learning (see “transfer learning” below) in which all or part of the weights of an artificial neural network pretrained on another task are further adjusted (“fine-tuned”) during the training on a new task
generalizability	in the context of ML models, generalizability refers to a model’s ability to perform well on new data not used during the training process
generative models	algorithms aiming to capture the data distribution of the training samples to be capable of generating novel samples resembling the data that they was trained on
inductive bias	set of assumptions of a model used for predictions over unknown inputs. for example, the model can be built such that it can only predict values within a certain range consistent with the expected range of values for a particular problem
learning rate	a parameter influencing the speed of the training of an ML model. a higher learning rate increases the effect of a single pass of the training data on the model’s parameters
loss function/cost function	a function used to evaluate a model during training. by iteratively minimizing this function, the model updates the values of its parameters. a typical example is mean-squared error of prediction
machine learning (ML)	machine learning, according to Mitchell, is the science that is “concerned with the question of how to construct computer programs that automatically improve with experience”. the terms ML and AI are often used interchangeably, but such usage is an oversimplification, as ML involves learning from data whereas AI can be more general
masking	deep learning paradigm for self-supervised learning. neural networks trained in a masked-modeling regime acquire powerful understanding of data by learning to reconstruct masked parts of inputs, *e.g.*, masked words in a sentence, numerical features artificially set to zeros, or hidden side-chains in a protein structure
multilayer perceptron (MLP)	a basic architecture of artificial neural networks. it consists of multiple fully connected layers of neurons. each neuron calculates a weighted sum of inputs from the previous layer, applies a nonlinear activation function, and sends the result to the neurons in the next layer
multiple sequence alignment (MSA)	a collection of protein or nucleic acid sequences aligned based on specific criteria, *e.g.*, allowing introductions of gaps with a given penalty or providing substitution values for pairs of residue types, to maximize similarity at aligned sequence positions. sequence alignments provide useful insights into the evolutionary conservation of sequences
one-hot encoding for protein sequence	each amino acid residue is represented by a 20-dimensional vector with a value of one at the position of the corresponding amino acid in the 20-letter alphabet and zeros elsewhere
overfitting	case of an inappropriate training of ML model in which the model has too many degrees of freedom, and it is allowed to use these degrees of freedom to fit the noise in the training data during training. as a result, the model can reach seemingly excellent performance on the training data, but it will fail to generalize to new data
regularization	in ML, regularization is a process used to prevent model overfitting. regularization techniques typically include adding a penalty on the magnitude of model parameters into the loss function to favor the use of low parameter magnitudes and thereby compress the parameter space. another popular regularization method for DL is to use a dropout layer, which randomly switches network neurons on and off during training
reinforcement learning (RL)	a machine learning paradigm in which the problem is defined as finding an optimal sequence of actions in an environment where the value of each action is quantified using a system of rewards. for example, if RL were used to learn to play a boardgame, the model would function as a player, the actions would represent the moves available to the player, and the game would constitute the environment. using game simulations, the model learns to perform stronger actions based on the “rewards” (feedback from the environment, *e.g.*, winning/losing the game) it receives for its past actions
self-supervised learning	a machine learning paradigm of utilization of unlabeled data in a setting of supervised learning. In self-supervised learning, the key is to define a proxy task for which labels can be synthetically generated for the unlabeled data, and then the task can be learned in a supervised manner. for example, in natural language processing, a popular task is to take sentences written in natural language, mask (see “masking” above) words in those sentences, and learn to predict the missing word. such a proxy task can help the model to learn the distribution of the data it is supposed to work with
semisupervised learning	a machine learning paradigm for tasks where the amount of labeled data is limited but there is an abundance of unlabeled data available. the unlabeled data are used to learn a general distribution of the data, aiding the learning of a supervised model. for example, all the data can be clustered by an unsupervised algorithm, and the unlabeled samples can be automatically labeled based on the labels present in the cluster, leading to enhancement of the data set for supervised learning, which can benefit its performance despite the lower quality of labeling
supervised learning	a machine learning paradigm in which the goal is to predict a particular property known as a label for each data point. for example, the datapoints can be protein sequences and the property to be predicted would be the solubility (soluble/insoluble). training a model in a supervised way requires having the training data equipped with labels
transfer learning	a machine learning technique in which a model is first trained for a particular task and then used (“transferred”) as a starting point for a different task. some of the learned weights can further be tuned to the new task (see “fine-tuning” above), or the transferred model can be used as a part of a new model that includes, for example, additional layers trained for the new task
transformer	transformers learn to perform complex tasks by deducing how all parts of input objects, *e.g.*, words in a sentence or amino acids in a protein sequence, are related to each other, using a mechanism called “attention”. the transformer architecture is currently one of the most prominent neural network architectures
underfitting	the case of an insufficient training of a ML model, where the model could not capture the patterns in the available data well and exhibits high training error. it can be caused for example by a wrongly chosen model class, too strong regularization, an inappropriate learning rate, or too short training time
unsupervised learning	a machine learning paradigm in which the goal is to identify patterns in unlabeled data and the data distribution. Typical examples of unsupervised learning techniques include clustering algorithms and data compression or projection methods such as principal component analysis. the advantage of these methods is the capability of handling unlabeled data, often at the expense of their predictive power

a Focusing on their meaning in the context of this Perspective. The terms from this table are highlighted in bold upon their first usage in the text.

**Figure 1:**
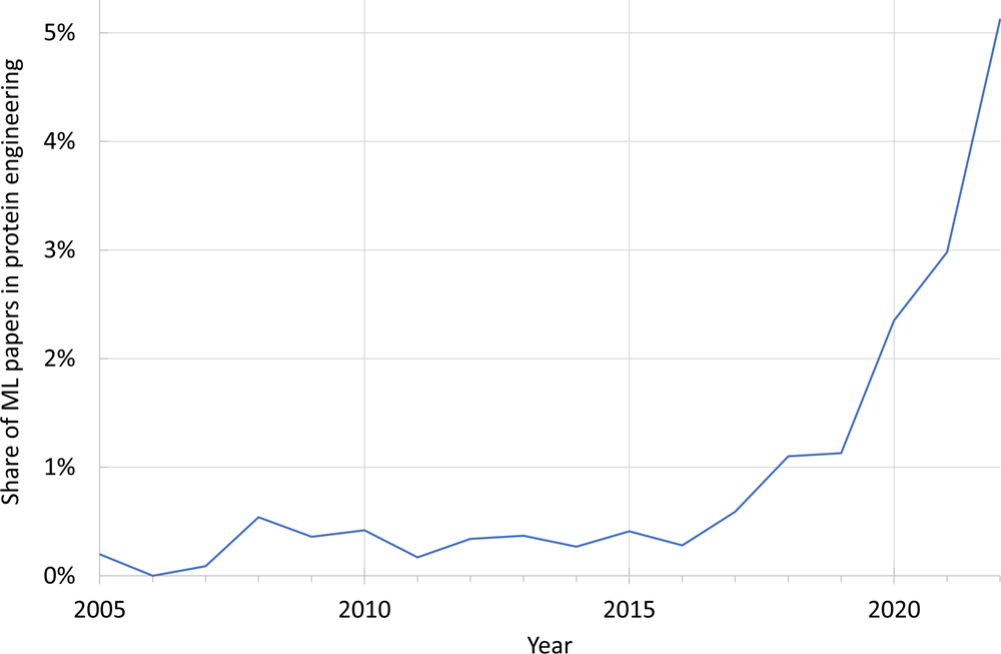
The trend in the use of machine learning in the literature on protein engineering. The graph shows the ratio of publications mentioning “machine learning” and “protein engineering” to all papers mentioning “protein engineering” in their title, abstract, or keywords, based on the Scopus database. This trend illustrates the increasing attention ML is receiving as a generally applicable and useful technology for protein engineering.

This paradigm shift is illustrated by the exponentially growing number of scientific articles describing the use of machine learning for protein engineering (Figure 1). The trend toward data-driven methods is expected to continue as technological advances allow us to accumulate, deposit, and reuse biological and biochemical data more effectively. This is being facilitated by initiatives such as the **FAIR principles**, which promote the findability, accessibility, interoperability, and reusability of data, and the European Open Science Cloud, which is designed to promote best practices in handling data.^12^ These large-scale initiatives are expected to accelerate the adoption of data-driven methods by making it easier for researchers to access, use, and share existing data and ensuring that these data are of high quality.

This Perspective focuses on the application of machine learning to protein engineering, which means improving the properties of biocatalysts by optimizing their sequences and tertiary structures using molecular biology techniques. Time-wise, we will primarily cover the period since our previous review on the same topic, published in 2019.^13^ As for particular areas, we will be focusing mainly on the applications regarding engineering by mutating known proteins rather than designing proteins *de novo*. While readers with a particular interest in *de novo* design might also find this Perspective helpful, as we cover many techniques common for various protein design tasks, for particular details on *de novo* design, such as deep **generative modeling**, we refer to other reviews.^14−16^ We will also introduce high-level concepts from machine learning to familiarize the reader with a broader context and will not cover in depth technical aspects such as specifics of various neural network architectures. We refer the readers to several excellent recent reviews on those topics.^10,17−23^ We will consider new methods from a user’s perspective. We find this important because the methods presented in research papers, while being exciting and innovative, often have only a limited impact if the wider community cannot quickly and easily adopt them. Moreover, we will draw inspiration from other domains, as we believe that identifying parallels between tasks in different fields can accelerate the development of more powerful and practically useful methods. In particular, by examining how solutions have been developed in other disciplines, we can learn effective ways of making methods and software tools applicable to a broader range of users.^24,25^

This paper is organized as follows. Section 2 briefly reviews the basics of machine learning and underlines the similarities and differences between data related to proteins and data in other domains. Section 3 provides a comprehensive review of machine learning in protein engineering and highlights recent progress in the field. In section 4, we examine a series of exciting recent case studies in which machine learning methods were applied to create new enzyme designs for use in the laboratory and in practical applications. In section 5, we identify gaps in the field that remain to be filled. Finally, in section 6, we investigate what inspiration we can draw from other disciplines to bring ML-based enzyme design to a new level.

## 2 Principles of Machine Learning

As some of our readers might be unfamiliar with machine learning (ML), we start with a brief introduction to the topic. We will cover the basics of ML-based pipelines and vocabulary, highlight similarities between protein engineering and other domains from a ML perspective, outline the main traits that distinguish protein data from other data types regularly used in ML, and summarize the challenges of performing ML with protein data.

### 2.1 Machine Learning Basics

Machine learning is often seen as a subcategory of **artificial intelligence** (AI). Its primary purpose is to learn patterns directly from available data and use the learned patterns to generate predictions for new data. Its main difference from other methods for modeling a system’s behavior, such as quantum mechanical calculations, is that ML does not rely on hard-coded rules to make predictions. Instead, ML models are mathematical functions that depend on generic parameters whose values are obtained (learned) through optimization using available data and an optimization criterion, the so-called **loss function**.

Since the final model is derived from the input data, careful data collection is vitally important for machine learning. In particular, any biases, measurement noise, and imbalances must be recognized and accounted for. Moreover, as ML is based on mathematical functions, every data point in a data set typically needs to be represented as a vector of numbers that are commonly referred to as **features**. Features may be obtained by simple encoding of the raw data, *e.g.*, **end-to-end learning** and **one-hot encoding**, but they may also represent more involved quantities derived from the raw data. For example, when predicting solubility from a protein sequence, the features may be simple amino acid counts, propensities of different residues to form secondary structures, conservation scores of proteins, or variables representing aggregated physicochemical properties.^26^ Choosing informative and discriminating features that provide relevant information about the underlying pattern in the data is crucial in ML because the features are the only data characteristics that the algorithm will exploit during training and when making predictions based on future inputs.

**Figure 2:**
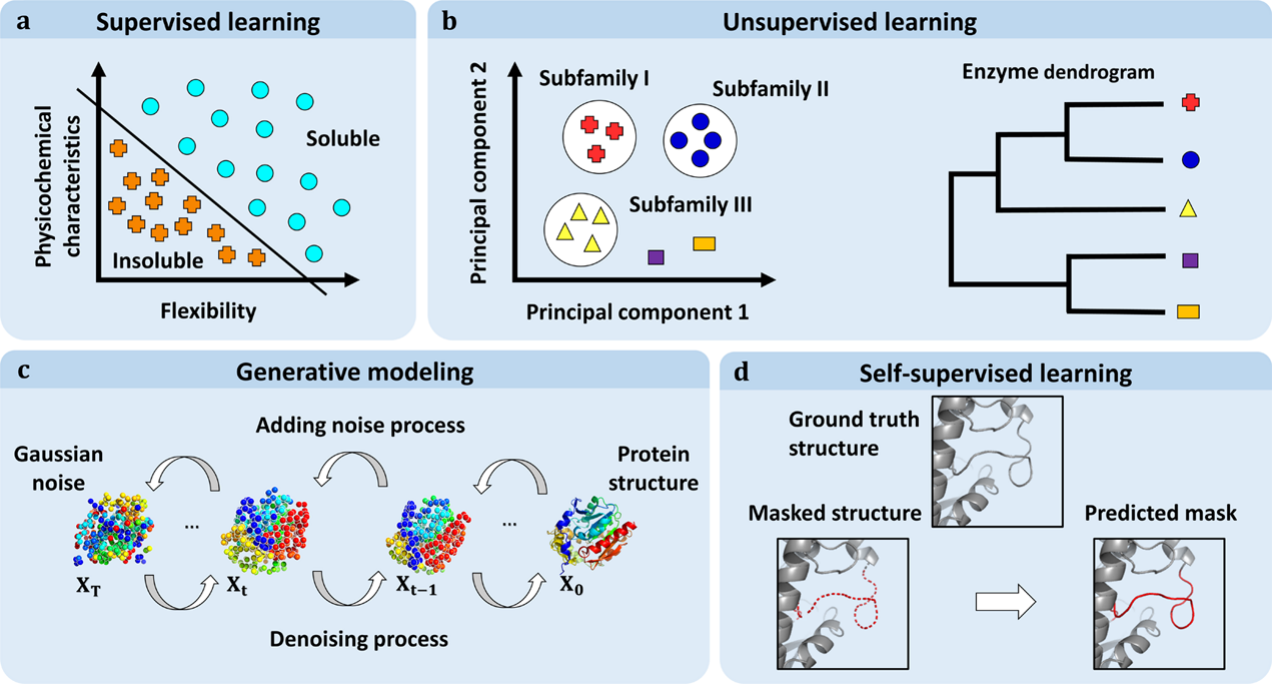
Four main categories of machine learning methods. (a) **Supervised learning** methods use labels. For example, each protein in a data set might be labeled “soluble” or “insoluble”, and the model would then aim to find the optimal decision boundary between these two classes in the feature space. The learned boundary is then used to make predictions about new data for which labels are unavailable. (b) **Unsupervised learning** methods typically find patterns, *e.g.*, clusters or groups) in unlabeled data. Examples include clustering enzymes into subfamilies or grouping them into a dendrogram. (c) **Generative models** learn the distribution of the training data to generate new instances corresponding to that distribution. Models of this type include diffusion models, which are trained to denoise synthetically noised inputs. The trained models can then be applied to random noise on the input to create a sample resembling the training data, *e.g.*, a new protein structure. (d) **Self-supervised learning** methods transform an unsupervised problem into a supervised problem, for example, by masking a part of a sequence or structure (red dashed loop) and then predicting the masked information (red loop). Such proxy tasks enable the model to learn important characteristics of the underlying data and can lead the model to perform well even on different tasks.

Several different categories of ML problems exist. In **supervised learning** problems, the goal is to predict a particular property (known as a label) for each data point (Figure 2A). For example, if we were seeking to predict protein solubility, each data point could be labeled “soluble” or “insoluble” based on experimental results. Data points can have multiple labels, so a given protein could have the labels “soluble”, “from a thermophilic organism”, and “globular”. Labels can form a set of classes or fall within a range of numerical values, giving rise to two subtypes of supervised learning problems: classification problems involving labels with no inherent order (*e.g.*, “soluble” or “insoluble”) and regression problems involving labels corresponding to numerical values (*e.g.*, protein yields). In contrast, the goal in **unsupervised learning** problems is to identify patterns in unlabeled data. Unsupervised learning techniques include clustering algorithms and data compression or projection methods, such as principal component analysis (Figure 2B). **Semisupervised learning** problems are those where the amount of labeled data is limited but there is an abundance of unlabeled data available. The unlabeled data are used to learn a general distribution of the data, aiding the learning of a supervised model. For example, all the data can be clustered by an unsupervised algorithm, and the unlabeled samples can be automatically labeled based on the labels present in the cluster, leading to the enhancement of the data set for supervised learning, which can benefit its performance despite the lower quality of labeling.

The boundary between supervised and unsupervised machine learning has been blurred by the emergence of methods that can create labels synthetically. For example, in a data compression method, the label might be the input itself and the algorithm may impose constraints (*e.g.*, a bottleneck in the architecture) that force the model to learn a more compact way of representing the data and their distribution. The algorithms that aim to capture the data distribution to generate new samples belong to a class of ML models called generative models. The most recent examples of this class include **diffusion models**, which have recently been used to generate protein backbone structures^27,28^ and predict the binding of a flexible ligand to a protein^29^ (Figure 2C). In diffusion models, synthetic training data are generated by gradually noising true data (*X*_0_) in a stepwise manner to ultimately obtain a maximally noised sample (*X*_T_). The sequences of increasingly noisy data are then reversed and used to train a model to denoise each individual step by having the (less noisy) sample *X*_*t*-1_ serve as a “label” for the (more noisy) following sample X_*t*_. For more details on diffusion generative models and their applications in bioinformatics, see the recent review.^30^ Alternatively, we can avoid the need for labels by **masking** a part of the input, *e.g.*, a residue in a protein sequence or structure,^31−33^ and training a model that will predict the masked part. In other words, the original data (*e.g.*, the amino acid that was masked) are treated as the label for the corresponding data point. Such approaches belong to the methods of **self-supervised learning** (Figure 2D) and are currently attracting considerable attention because of their great success in large language models; it turns out that this “self-supervision” approach allows algorithms to learn useful characteristics of the data, such as grammar and semantics in the case of natural language models. The following sections present some applications of self-supervised learning in the enzymology domain.

In supervised learning, **underfitting** and **overfitting** are two critical concepts that must always be considered. Underfitting refers to a situation in which either the selected class of models is insufficient to approximate patterns in the available data, the **regularization** is too strong, or the parameters of the training process such as the duration of the training or the **learning rate** were inappropriate. As a result, the model fails to capture the relationship between the input and output and has a high training error. Conversely, overfitting happens when a model has too many degrees of freedom, allowing it to start fitting noise in the training data during the training process. This leads to poor **generalization** and a significant drop in performance when the model is applied to new inputs. Robust evaluation of trained models is therefore crucial in machine learning to obtain feedback on the training process and develop improved training protocols or model hyperparameters.

The best practice in machine learning is to split the available data into three disjoint subsets: a training set, a validation set, and a test set. A model learns the underlying patterns within the data by fitting its parameters to the training set. The validation set is provided to the model at certain stages of the training for basic evaluation, and the results of these evaluations are used to select the model’s hyperparameters. Finally, the test set is used to get a realistic estimate of the model’s performance and is therefore only used once training has been completed and the final values for the hyperparameters have been set. Since the model does not see the test set during the training process, the model’s performance, when applied to this “new” data set, should be similar to that achieved with the test set if the test set accurately represents the general distribution of the studied data.

The choice of evaluation metric depends on the task at hand. Classification problems are mainly evaluated based on **model accuracy**, *i.e.*, the ratio of correct predictions to the number of total predictions. Regression problems are typically evaluated based on the difference between the predicted and ground truth labels, so popular metrics include measures of the correlation between these labels as well as the mean squared error (MSE) and related metrics such as the root-mean-square error (RMSE). More complex problems often require customized metrics. For instance, in a protein structure prediction task, one might use the MSE between the predicted and actual locations of Cα atoms, which can be expressed using a fixed coordinate system (global alignment) or in terms of the local coordinates of each residue (local alignment). Both metrics indicate how well a predicted structure aligns with the corresponding actual structure.

### 2.2 Parallels between Machine Learning Tasks in Biocatalysis and Those in Other Domains

One of the strengths of machine learning is its universality, as the algorithms used for protein engineering tasks are similar to those used in other domains. Therefore, scientists working with protein data can reuse and build upon existing solutions from other fields, such as natural language processing, computer vision, and network analysis.

Natural language processing (NLP) is a field of computer science that aims to teach computers how to understand and handle natural languages. New ML techniques have enabled great advances in this field in recent years. For instance, a common task in NLP is to generate semantically and grammatically correct sentences. In protein engineering, the strings of amino acids representing primary sequences can be regarded as words constructed from an alphabet of twenty letters representing the canonical amino acids. These words can represent secondary structures or other motifs that can be combined in meaningful ways to create sentences in the language of protein structure that correspond to functional proteins. Another common task in NLP is the assignment of labels to individual words (*e.g.*, to predict lexical categories or identify relevant information) or phrases (*e.g.*, for sentiment analysis). This data structure resembles that of annotated protein data sets with labels representing protein stability, binding affinity, specificity, or other characteristics.^34^ Moreover, the complexities of the relationships between protein sequence, structure, and function are reminiscent of those in human languages, prompting researchers to adapt **transformer**-based large language models used in NLP to protein engineering tasks.^35^

Another field of computer science that has benefited greatly from recent advances in ML is computer vision, and techniques developed for use in this field have also found applications in the study of the protein structure. For example, protein structures can be converted to arrays of voxels (3D pixels) via the application of a discrete grid. The resulting representations are similar to those of volumetric 3D images, enabling the application of ML architectures, such as convolutional neural networks, that were originally designed to process image data. These networks learn representations through convolution and hierarchical aggregation and have recently been used to predict protein mutation landscapes,^31^ protein–ligand binding affinity,^36^ and the interactions of proteins with water molecules.^37^ Denoising diffusion probabilistic models are another class of computer vision models that have been applied in protein structure prediction. They are trained to denoise existing samples, which allows them to generate novel samples by morphing random noise. This has led to major breakthroughs in image generation and the emergence of the highly successful models, such as DALL-E 2^38^ or Stable Diffusion.^39^ In protein science, such models have been used to perform fast protein–ligand binding,^29^ generate new small-molecule ligands^40^ and linkers,^41^ and perform *de novo* design of large proteins.^27,28^

The parallels between images and protein structures can be further exploited by adapting techniques developed for video analysis to predict protein dynamics. For example, a trajectory generated in a molecular dynamics simulation can be regarded as a temporal sequence of 3D images. This makes protein dynamics analysis similar to video processing and implies that video methods for event detection^42^ can be applied to molecular dynamics trajectories to detect events such as the opening of a tunnel.^43^ Video processing techniques can thus be adapted to clarify a protein’s function by analyzing the movement of individual atoms or groups of atoms within a protein structure in a manner similar to the movement of objects or groups of objects in a video. Moreover, there have been remarkable advances in ML techniques for video synthesis^44−46^ that could inspire new methods for capturing and synthesizing protein dynamics.

Finally, one more domain is relevant to protein engineering: network analysis, which involves studying the properties and structures of interconnected elements. Network analysis techniques have been used successfully to study diverse social and biological networks, including the development of Covid-19-related sentiment on social networks^47^ and protein–protein interaction networks.^48^ Interactions between proteins or between a protein and a ligand can be represented as networks (graphs) in which nodes correspond to proteins and ligands while edges correspond to biological relationships between them. Once such a network has been defined, link prediction or community detection methods can be applied.^49^ Alternatively, a protein structure can be represented as a network where nodes correspond to residues or individual atoms and edges correspond to inter-residue interactions or interatomic bonds. This makes it possible to use graph-based ML algorithms for tasks such as predicting protein function, solubility, or toxicity.^50,51^ Moreover, data on protein interactomes and the structures of small molecules have been used to drive theoretical research on graph-based ML: the best-established benchmark for graph learning, OGB, includes multiple biochemical data sets of this type.^52^

### 2.3 Challenges of Machine Learning for Protein Data

As discussed above, there are striking similarities between protein engineering tasks and other ML domains, including natural language processing, computer vision, and network analysis. However, protein data also present unique challenges related to the representation of proteins, the construction of labeled data sets, and the establishment of robust training protocols.

The choice of protein representation is a key step in all protein-related computational tasks. Proteins can be represented at different levels of detail, from a discrete and accurate 1D representation of their amino acid sequence to a continuous and less accurate 3D representation of every atom position, including or excluding chemical bonds. The selected representation determines the type and amount of information available to the computational model and the range of applicable model architectures.

The go-to representation of a protein sequence is a string (word) constructed using an alphabet of 20 amino acids (letters). The length of the string equals the number of residues, and the *n*^th^ character encodes the amino acid at the *n*^th^ position in the protein sequence. *In silico*, the amino acids are typically represented using one-hot encoding. When the amount of data available for training is not enough for end-to-end learning, one-hot encoding of sequences can further be transformed into values corresponding to specific physicochemical characteristics of amino acids, *e.g.*, using AA indices.^53^ These indices provide additional information and interpretability to the pipeline, although they were shown to perform on par with random vectors for some tasks.^54,55^ Another strategy to enrich the protein sequence representation is to include evolutionary information using a **multiple sequence alignment** (MSA) instead of a single protein sequence. This evolutionary information is valuable in various tasks, most notably in structure prediction because the covariance of different residue positions in the sequence can be related to the residues’ spatial proximity.^56^

The options for representing a protein’s structure are more varied; representations may include all atoms, only selected chemical elements (*e.g.*, all atoms except hydrogens), or just key components of residues (*e.g.*, the α-carbons). Moreover, they may include different types of information about these atoms and/or residues. Ideally, to enable data-efficient model training, structural representations should be invariant to rotation, translation, and reflection. However, straightforward representations based on the 3D coordinates of the residues (atoms) lack this property. It is therefore common to represent protein structure using an inter-residue or interatomic distance matrix in which each row and column is assigned to a specific residue (or atom) of the protein and the value of each matrix entry is equal to the distance between the corresponding residues (atoms). Such a matrix is necessarily symmetric; therefore, it is common to take the upper (or lower) triangular part and convert it into a 1D vector for processing, *e.g.*, by a neural network. While this representation is rotationally and translationally invariant, it is also inherently redundant, as its spatial complexity is quadratic with respect to the number of residues (atoms).

Graph-based protein representations have recently attracted considerable attention.^57^ A graph consists of a set of nodes linked by a set of edges. The nodes typically represent residues, atoms, or groups of spatially close atoms, while the edges usually correspond to chemical bonds, spatial proximity (contacts) between the nodes, or both.^58^ Graph-based protein representations are very flexible because the definition of the nodes and edges can be tailored to specific tasks and they can be made **equivariant** to rotation, translation, and reflection.^59^ A convenient definition of the nodes and edges can also introduce an **inductive bias** that improves the model performance. For example, edges corresponding to chemical bonds can guide a model toward learning chemical knowledge more rapidly or with less data than would otherwise be needed. Graph neural network (GNN) architectures have recently achieved state-of-the-art performance in multiple protein-related tasks, as exemplified by the DeepFRI^60^ and HIGH-PPI^61^ methods for predicting protein function and protein–protein interactions, respectively. Special graph-based protein representations, such as point clouds (no edges) or complete graphs (a full set of edges), are especially convenient for processing using powerful transformer models.^62,63^

A more general approach to protein representation is to directly learn the representation by a deep learning model, a direction that is currently on the rise in biology.^64^ Its aim is to remove the suboptimality of human-made choices by inferring the representation parameters from existing data. Furthermore, it is often possible to learn such a representation through self-supervised training, *i.e.*, without the need for annotated data. These representations can be obtained from the sequence data, *e.g.*, as done by the ESM (“Evolutionary Scale Modeling”) language models,^65−67^ as well as from large structural data sets, *e.g.*, as done by GearNet.^68^ More and more models combine both sources of data, *e.g.*, ESM-GearNet.^32^

Obtaining appropriately labeled data sets can be challenging when seeking to apply ML in enzyme engineering because there is often a trade-off between data quality and quantity when selecting methods for acquiring experimental data. Most reliable biochemical methods using purpose-built instruments can only provide data for small numbers of protein variants^69^ and are thus generally insufficient for representative sampling of vast mutational spaces. Conversely, high-throughput methods such as Deep Mutational Scanning (DMS) are prone to data quality issues^69^ and face a throughput bottleneck in the case of enzymes whose screening is considerably slower than sequencing.^70^

The process of compiling new data sets from multiple sources can be complicated by the inconsistency of conventions in protein research. These inconsistencies include the differing biases of various experimental tools, the use of different distributions for data normalization, and inconsistent definitions of quantities such as stability, solubility, and enzyme activity. All of these can introduce errors into constructed data sets, for example, by causing contradictory labels to be applied to the same protein. Protein data may also contain biases introduced by the design strategy. For example, alanine tends to be overrepresented in mutational data due to the widely used alanine scanning technique.^71^ It is important to consider these biases during data set construction and when interpreting model outputs since the composition of the training set significantly affects the space of patterns explored by the model.

A significant amount of protein data has been gathered over the years. However, much of these data are proprietary and thus inaccessible to the academic community. In addition, publicly available data sets are often published in an unstructured way, which limits their usability. While large language models such as GPT-3,^72^ GPT-4,^73^ or BioGPT^74^ are remarkably effective at summarizing text on large scales, mining relevant data from publications still requires considerable human effort.

Some ML packages, *e.g.*, TorchProtein,^75^ offer preprocessed data sets for various protein science tasks, making protein research more accessible to ML experts from other domains. Other packages, such as PyPEF^76^ provide frameworks for the integration of simpler ML models together with special encodings derived from the AAindex database of physicochemical and biochemical properties of amino acids.^53^ Despite this progress, the development of ML models for protein data requires a certain level of biochemistry domain knowledge to account for the specifics of protein data absent in other application areas of ML. These specifics include the evolutionary relationships and structural similarities between proteins. Models produced without the benefit of such expertise may lack practical utility due to data handling errors.

One common type of data handling error is data leakage between data splits. The training, validation, and test sets should not share the same (or nearly the same) data points because such overlaps could lead to over estimating the model’s performance on new data, compromising the model’s evaluation. In some data sets, all of the data points are distinct enough that randomly splitting the available data into disjoint sets is a viable strategy. However, more complex splitting strategies are often needed when dealing with protein data to avoid problems such as evolutionary data leakage.^77^ It may also be important to consider multiple levels of separation when dealing with protein data, *e.g.*, consisting of mutations and their effects. For example, one might want to ensure that the same substitutions, positions, or proteins do not appear in the training and test sets. Defining protein similarity is also a challenging task for which multiple strategies exist. Many of these strategies involve clustering proteins based on sequence identity or similarity thresholds and then ensuring that all members of a given cluster are assigned to the same set when splitting the data. This strategy is particularly useful for constructing labeled data sets of protein structures because such data sets are primarily sourced from large redundant databases such as PDB^78^ (see Table S2). However, clustering in the sequence space can be insufficient in some cases. For example, distantly related proteins may have very similar active site geometries even though their sequence homology is low.^79^ Clustering may thus be performed at the level of the structural representation instead. While such strategies have only rarely been used in the past, they may become more common due to the emergence of new tools for protein structure searching such as Foldseek.^80^

## 3 Protein Engineering Tasks Solved by Machine Learning

This section provides a brief overview of the protein engineering tasks that have already drawn the attention of machine learning developers. The first of these tasks is functional annotation, which is important because the overwhelming majority of sequences in protein databases remain unannotated and *in silico* prediction is necessary to keep pace with the exponentially growing number of deposited sequences. We will then cover the available labeled data sets and state-of-the-art ML tools for predicting mutational effects in proteins, as well as strategies for protein design based on their predictions. In addition, we will review published methods for leveraging unlabeled protein sequence and structure data sets to help guide protein engineering. Finally, we conclude with examples showing how ML models of protein dynamics can facilitate the selection of promising mutations.

### 3.1 Functional Annotation of Proteins

Knowledge of protein functions is fundamental for protein engineering pipelines. For instance, in protein fitness optimization, scientists start from a characterized wild-type sequence with some degree of the desired function.^81−83^ Likewise, in biocatalysis, one needs to know the function of enzymes to assemble a biosynthetic pathway from plausible enzymatic reactions.^84,85^

Traditionally, scientists have characterized the functions of proteins via laborious, time-consuming, and costly wet lab experiments. However, owing to high-throughput DNA sequencing, the exponentially growing repertoire of protein sequences is reaching numbers far beyond the capabilities of experimental functional annotation; for example, the Big Fantastic Database (BFD, Table S2) contains 2.5 billion sequences to date. Functional annotation is particularly important for enzymes. A broad-level annotation (*e.g.*, enzyme family) can be achieved relatively easily by sequence homology and by searching for protein domain motifs, but detailed annotation of enzyme substrates and products currently requires experimental characterization. To accelerate this process, a substantial recent effort has been dedicated to the development of novel computational methods for functional annotation.

The developed computational methods heavily rely on data sets of previously characterized enzymatic functions for training. For example, information on protein families and domains is often sourced from the Pfam,^86^ SUPERFAMILY,^87^ or CATH^88^ databases. Enzymatic activity data often come from databases such as Rhea,^89^ BRENDA,^90^ SABIO-RK,^91^ PathBank,^92^ ATLAS,^93^ and MetaNetX.^94^ The ENZYME database under the Expasy infrastructure^95^ provides the Enzyme Commission (EC) numbers, the most commonly used nomenclature for enzymatic functions. EC numbers are a hierarchical classification system categorizing enzymatic reactions at four levels of detail, with the fourth level being the most detailed. An EC number groups together proteins with the same enzymatic activity regardless of the reaction mechanisms.^96^ The data from the above-listed databases have also been post-processed and organized into data sets such as ECREACT^97^ or EnzymeMap,^98^ which should further facilitate the development of computational models.

The models for enzymatic activity prediction typically use enzyme amino acid sequences as input, as the goal is to directly annotate the outputs of high-throughput DNA sequencing. The incorporation of the structural inputs, facilitated by the recent breakthroughs in structure prediction,^67,99^ is still to be explored more by the community. The outputs of these models generally fall into three categories based on the resolution of predictions. First, the most general models predict protein families and domains. Second, the EC class-predictive models provide a more detailed estimation of enzymatic activity. Finally, the most comprehensive picture of enzymatic activity requires models for predicting an enzyme’s substrates and corresponding products. Several recent deep learning models predict protein families and domains.^100,101^ Although such models are crucial for studying proteins, they have only a limited applicability in enzymatic activity prediction, as a single protein family can combine enzymes catalyzing different reactions.^102^ To meet the needs of protein engineering, the models predicting the EC numbers appear to be more relevant, as they can capture the catalytic activities.

Over the years, the community attempted to predict EC numbers using multiple sequence alignment and position-specific scoring matrices (PSSM) or hidden Markov model (HMM) profiles,^103−105^*k*-nearest neighbor-based classifiers,^104−108^ support vector machines (SVM),^105,109−112^ random forests,^113,114^ and deep learning.^60,115−119^ Most methods approached the prediction of EC numbers as a classification problem, which led to poor performance on the under-represented EC categories. The recent deep-learning-based method^119^ tackled the EC number prediction via a contrastive learning approach, training a Siamese neural network on top of sequence **embeddings** from a pretrained protein language model.^66^ The resulting predictive algorithm, CLEAN, can better identify enzyme sequences that belong to any EC category, including underrepresented ones. CLEAN achieved state-of-the-art performance in EC number prediction *in silico*, and it was experimentally validated *in vitro* using high-performance liquid chromatography–mass spectrometry coupled with enzyme kinetic analysis on a set of previously misannotated halogenase enzyme sequences.

EC class prediction enables downstream applications such as retrobiosynthesis.^85^ For instance, planning of biosynthesis has been tackled by predicting the chemical structure of the substrate and the required enzyme EC number from the provided enzymatic product using a transformer-based neural network.^97^ Several other published deep learning models aspired to estimate the substrate of an enzymatic reaction based on its product.^120,121^ Such models can be used to prioritize enzyme selection based on the EC class or the substrate/product pair. However, the assignment of a specific enzyme sequence (*i.e.*, not only the EC class) to a desired reaction remains a challenge for future development.

Recently, the first general models predicting interaction between individual enzymes and substrates/products were published.^122,123^ These DL-based models take pairs of a protein sequence and a small molecule as inputs and predict their possible interaction. Unfortunately, none of the general substrate–enzyme interaction models were validated in wet lab experiments. Furthermore, in Kroll et al. the authors admit poor generalization of the model to out-of-sample substrates.^123^ Moreover, enzyme-family-specific models were shown to outperform the general models in predicting the enzyme–substrate interactions.^124^ To sum up, the practical applicability of general enzyme–substrate interaction models has yet to be determined.

### 3.2 Supervised Learning to Predict the Effects of Mutations

The ability to predict mutational effects on various protein properties, such as solubility, stability, aggregation, function, and enantioselectivity, is another desirable goal of protein engineering. From the machine learning perspective, this implies having a model that takes a reference protein and its variant as the input and predicts the change in the studied property as the output. Intuitively, this could be achieved by supervised learning on the labeled data sets of wild-type proteins first (*e.g.*, to predict a solubility score, binding energy, or melting temperature of a given protein), applying the trained model to independently predict labels of the reference protein and its variant, and then taking the difference between the two predicted scores. The attractiveness of this strategy comes from large annotated data sets of wild-type proteins available for training. For example, the Protein Structure Initiative^125^ generated a massive data set TargetTrack, often used for protein solubility prediction.^26^ Additionally, the more recent Meltome Atlas of protein stability obtained by liquid chromatography-tandem mass spectrometry^126^ was used for predicting melting temperatures.^127^ Our ongoing effort to predict highly valuable melting temperatures solely from the protein sequence resulted in the development of the TmProt software tool (https://loschmidt.chemi.muni.cz/tmprot/). Large labeled data sets usually provide enough training data for powerful end-to-end deep learning.^34,128,129^ Nonetheless, when the training data set does not contain mutations, a few substitutions will usually result in similar predicted labels (*e.g.*, solubility scores), in contrast to dramatic changes often observed in experiments. Therefore, the strategy of taking the difference between the predicted labels for a reference protein and its variant typically fails to produce reliable predictors for mutational effects.^130^

A more promising route is to use labeled mutational data sets for training. This strategy has its own limitations, since such data sets are not only scarce but also sparse in terms of the extent of the mutational landscape that is probed (the sequence space grows exponentially with the number of mutated residues) and biased toward several overrepresented proteins.^13,131^ These barriers severely hinder the use of ML, which relies heavily on the availability of good quality data with a high coverage of the space of interest. Therefore, additional data curation and processing, adjustments to training protocols, and more thorough and critical data evaluations are typically needed. Such efforts will be a crucial first step in establishing reliable ML pipelines for predicting mutational effects.

The most abundant and diverse mutational data come from general biophysical characterizations that are performed routinely in most protein engineering studies, including measurements of protein expressibility, solubility, and stability. The major challenge when using such data lies in collection and curation: measurements are scattered across the literature and often reported *ad hoc* because they are generally complementary to a study’s main results.^132^ This highlights the importance of establishing and maintaining databases with protein annotations to facilitate data discoverability and reuse. For example, we recently released SoluProtMutDB,^133^ which currently has almost 33 000 labeled entries concerning mutational effects on the solubility and expression of over 100 proteins. This database incorporates all of the data points that were recently used to develop solubility predictors (Table 2), which achieved correct prediction ratios of around 70%.^133^

**Table 2:** Recent Applications of Supervised ML Tools in Prediction of the Effects of Mutations in Protein Engineering^a^

targeted property	tool	training data	method	input	Web site
stability (ΔΔG)	BayeStab (134)	2648 single mutations from 131 proteins (S2648) derived from ProTherm (135)	Bayesian neural networks	PDB files with the WT and mutant structures	http://www.bayestab.com/
	PROST (136)	2647 single mutations from 130 proteins (S2648) derived from ProTherm (135)	ensemble model	sequence (FASTA) plus a list of mutations	https://github.com/ShahidIqb/PROST
	KORPM (137)	2371 single mutations from 129 proteins, derived from ThermoMutDB (138) and ProTherm (135)	nonlinear regression	a list of PDB files and single-point mutations	https://github.com/chaconlab/korpm
	ABYSSAL (139)	376 918 single mutations from 396 proteins (140)	siamese deep neural networks trained on ESM2^67^ embeddings	ESM2 embeddings of the WT and mutant sequences	https://github.com/dohlee/abyssal-pytorch
solubility	PON-Sol2 (141)	custom data set: 6328 single mutations from 77 proteins	LightGBM	a list of sequences (FASTA) plus lists of single-point mutations	http://139.196.42.166:8010/PON-Sol2/
activity	DLKcat (142)	custom data set: 16 838 data points from BRENDA (90) and Sabio-RK (91)	graph-based and convolutional neural networks	a list of substrate SMILES and enzyme sequences	https://github.com/SysBioChalmers/DLKcat
	MaxEnt (143)	various custom MSAs and kinetic constant data sets	statistical Potts model	single and pairwise amino acid frequencies from MSA	https://github.com/Wenjun-Xie/MEME
	MutCompute (31)	19 436 protein structures	self-supervised convolutional neural network	protein structure	https://mutcompute.com/
	innov’SAR (144)	custom data set: 7 variants vs 3 substrates at two pH levels	partial least-squares regression	N/A	N/A
	ML-variants-Hoie-et-al^145^	custom data set: over 150 000 variants in 29 proteins	random forest	Custom preprocessing, derived from PRISM approach (146)	https://zenodo.org/record/5647208
					https://github.com/KULL-Centre/papers/tree/main/2021/ML-variants-Hoie-et-al
optimal catalytic temperature *T*_opt_	ML-guided directed evolution of a PETase (147)	custom data set: 2643 enzymes from BRENDA^90^	random forest	N/A	N/A
	TOMER (148)	custom data set: 2917 enzymes from BRENDA^90^	bagging with resampling	a list of sequences (FASTA)	https://github.com/jafetgado/tomer
substrate specificity	ML-guided directed evolution of an aldolase^149^	131 experimentally characterized variants	Gaussian process	N/A	N/A
protein aggregation	ANuPP^150^	1421 hexapeptides obtained from CPAD 2.0,^151,152^ WALTZ-DB,^153,154^ and AmyLoad^155^ databases	ensemble of 9 logistic regressors	a list of sequences (FASTA)	https://web.iitm.ac.in/bioinfo2/ANuPP/about/
	AggreProt	1416 hexapeptides obtained from WALTZ-DB^153,154^	deep neural network	up to 3 sequences (FASTA) and (optionally) 3D structures	https://loschmidt.chemi.muni.cz/aggreprot
binding affinity (ΔΔG)	GeoPPI (156)	SKEMPI 2.0 (157)	graph neural network and random forest	PDB file, the names of interacting chains, and mutations	https://github.com/Liuxg16/GeoPPI

a N/A = not available.

Protein stability measurements are another type of widely available biophysical data that can be used in ML. Protein stability is typically quantified in terms of the melting temperature (*T*_m_) or the Gibbs free energy difference between the folded and unfolded states (ΔΔ*G*). Several protein stability databases exist, including FireProtDB,^158^ ThermoMutDB,^138^ and ProThermDB,^135^ and their data have often been used to train ML predictors that have achieved Pearson’s correlations of up to 0.6 and RMSE values of 1.5 kcal/mol when applied to independent test sets.^159^ Interestingly, these numbers have barely changed over the past decade, indicating that a qualitative paradigm shift might be needed to advance ML-based prediction of mutation-induced protein stability changes.^132^ It is possible that large new data sets could provide the necessary boost, and some exciting studies collecting such data are already appearing: cDNA display proteolysis was recently used to measure the thermodynamic stability of around 850 000 single-point and selected double-point mutants of 354 natural and 188 *de novo* designed protein domains between 40 and 72 amino acids in length.^140^

Changes in catalytic activity upon mutation also attract the attention of ML researchers. Predicting mutational effects on enzyme activity is more challenging than predicting protein stability and solubility due to the enormous diversity of enzymatic mechanisms. One rich source of such mutational data is large-scale deep mutational scanning.^69^ These experiments combine high-throughput screening and sequencing and typically score protein variants by comparing their abundance before and after a specific selection is applied. These data sets provide comprehensive overviews of the local mutational landscapes of various enzymes and are of significant value for ML due to their unbiased mutant coverage. Several groups have already assembled various deep mutational scanning (DMS) data sets for benchmarking effect predictors,^145,160−164^ and we expect this trend to continue as more data sets appear. Notable works of this type include the recently published activity landscapes of the phosphatase,^165^ dihydrofolate reductase,^166^ DNA polymerase,^167^ and palmitoylethanolamide transferase.^168^

DMS can be applied to a wide range of enzyme functions due to its high flexibility with respect to selection procedures. However, its high throughput comes at the cost of limiting the number of protein targets that can be used in a study; often, only a single case is examined. The desire to target multiple enzymes simultaneously motivated the creation of another notable database of enzyme activity changes: D3DistalMutation.^169^ It contains data derived from UniProt annotations representing over 90 000 mutational effects in 2130 enzymes. However, its potential in ML has not yet been explored.

Other protein characteristics may also be used as targets for protein engineering and machine learning. These targets are often selected based on the enzyme of interest and may include important functional traits such as substrate specificity,^149^ enzyme enantioselectivity,^170,171^ kinetic constants,^142−144^ temperature sensitivity,^172^ or temperature optima.^147,148^ In addition, several mutational data sets that can be used for ML-based tools focusing on protein folding rates, binding, and aggregation have been deposited in the VariBench benchmark data set.^173^ Selected recent examples of these tools are listed in Table 2. An overview of the described databases and data sets is given in Table S2.

### 3.3 Approaches to Design Mutations

While tools for predicting effects of mutations have become increasingly advanced in recent years, in their simple form, they can only provide labels for a given substitution. However, the desired outcome of protein engineering pipelines is to have a list of promising protein variants for experimental validation. Therefore, even if a reliable ML-based tool for predicting effects of substitutions is available, the problem of suggesting promising hypothetical designs must be addressed. This problem may become a major bottleneck, since even if the prediction of single- or multiple-point mutational effects is fast, evaluating all possible combinations of mutations remains unfeasible. Therefore, there is a growing need for tools that simultaneously predict the effects of mutations and reduce the search space, which is the focus of this subsection.

A major challenge in the development of such tools is finding ways to efficiently reduce the space of multipoint mutants. In an analysis of nine case studies, Milton and coauthors found that the effects of half of the multipoint mutations influencing enzymatic properties could not be predicted using knowledge of the corresponding single-point mutations, with the associated complexities resulting from direct interactions between residues in some cases and long-range interactions in others.^174^ This common nonadditive behavior, which is known as epistasis, has prompted the development of ML models and combinatorial optimization algorithms capable of scoring or searching multipoint mutants by design. Several approaches discussed below have been proposed to overcome this challenge, more on this topic can be found in a review.^175^

One such approach is to produce a library of variants for screening using reliable physics- and evolution-based tools. Even a time-consuming preselection of promising hotspots can drastically reduce the computational time of the downstream ML scoring and search.^176^ For example, HotSpot Wizard 3.0^177^ achieves robust selection of hotspots by using a number of sequence and structure-based filters to identify mutable residues for which mutation effects are then quantified using the well-established Rosetta and FoldX tools. Another example is the FuncLib web server, which computes promising single-point active-site mutations using evolutionary conservation analysis and Rosetta-based stability calculations.^178^ This tool exhaustively models each combination of mutants and ranks them by energy. Evolutionary information can also be captured by ML-based models^179^ and used to suggest promising substitutions, *e.g.*, amino acids with conditional likelihoods higher than the wild-type.^180^

**Figure 3:**
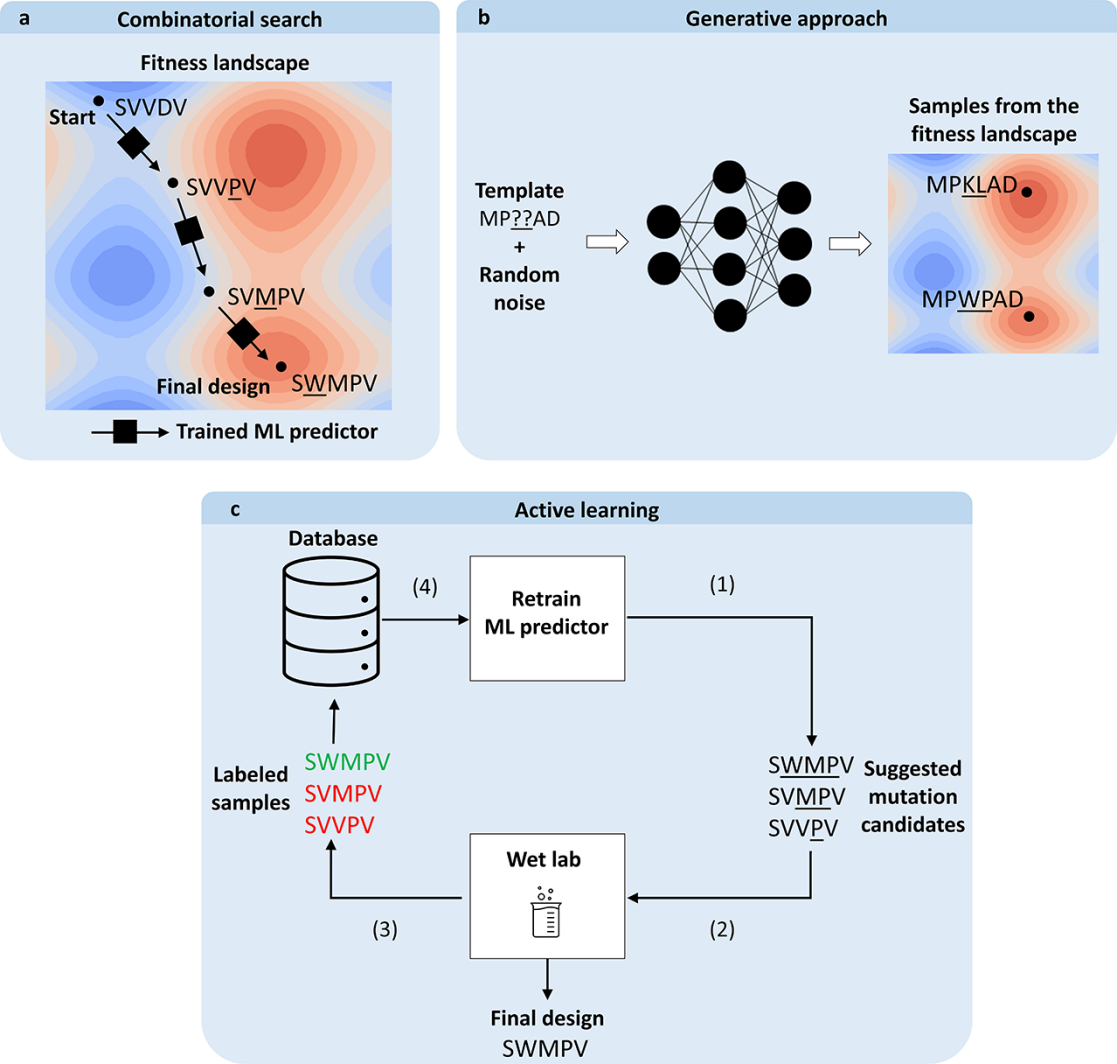
Selected ML strategies for designing new enzyme variants. (a) *In silico* evolutionary combinatorial search for favorable mutations. A machine learning predictor is used to iteratively evaluate candidate mutations for some desired property, *e.g.*, stability. The mutated amino acid in each step is underlined. (b) Generation of favorable mutants. The machine learning tool directly infers the sequence with high property values (*e.g.*, stability) from a fitness landscape learned and captured in the weights of the model during the training. (c) An active learning loop: an ML predictor from (a) or (b) is used to (1) propose enzyme variants that are (2) evaluated experimentally, and the resulting data are used to (3) update the knowledge database. The ML model is then (4) retrained on the updated knowledge database, and new variants are designed.

Mathematical optimization methods can generate promising protein sequence candidates *in silico* by iteratively producing new designs based on available ML scoring data (Figure 3). One group of such methods uses the ML predictor as a black-box oracle to evaluate existing candidates. This evaluation is then used to approximate the “fitness” of sequences, which is in turn used to navigate the sequence landscape and generate a new set of candidates using tools such as evolutionary algorithms^82,181^ or simulated annealing.^182^ However, approximating complex mutational landscapes using oracles representing estimated, simplifying distributions can harm the optimization process and prevent the optimal solution from being found.^183^ Adaptive sampling of the design space can be used instead to obtain better results.^81^ Other alternatives are to use generative models^184^ or rely on so-called white-box optimization, which involves using knowledge of a predictor’s internal workings to find the optimal solution. For example, linear regression coefficients can be used to suggest modifications (mutations) of the input that alter the corresponding features in the desired direction. White-box methods are discussed further in the context of **explainable AI** in section 6.1.

While *in silico* optimization methods enable the iterative generation of promising designs, they rely entirely on the ability of a predictor to correctly score any given point in a mutational landscape, which might be an unrealistically strong assumption. A possible alternative (albeit one that is more costly and has lower throughput) is to directly incorporate experimental validation into the optimization loop. This experimental input can guide search algorithms to more promising parts of the mutational landscape in a manner that is akin to directed evolution. While advanced search methods of this type have previously been used to improve traditional directed evolution,^82,182^ they do not fully exploit the potential of experimentally characterizing intermediate variants. ML-based **active learning** methods accelerate directed evolution by iteratively extracting knowledge from all characterized variants and selecting the most promising ones.^185^ By relying on the new experimental data, only a limited number of training samples can be expected,^186^ confining the choice of ML models to those with a lower number of parameters, such as **multilayer perceptrons** (MLP) with as few as two layers.^187^ A recent advance in the area of active learning is the development of GFlowNets,^188^ networks designed to suggest diverse and accurate candidates in a machine–expert loop to accelerate scientific discovery. Studies using such networks have demonstrated their potential for designing small molecules;^189^ however, the utility for the design of proteins remains to be reliably demonstrated.

Powerful design techniques for the exploration of variants of enzymes and other proteins often rely not only on combining experimental and *in silico* techniques but also on combining multiple *in silico* approaches and sometimes different ML techniques. For example, focused training ML-assisted directed evolution (ftMLDE)^186^ combines unsupervised and supervised training approaches by using unsupervised clustering to construct a training set for supervised classifiers. These classifiers are then used to select promising mutants with tools such as CLADE 2.0.^190^ Similarly, unsupervised learning on millions of sequences was used to obtain a protein representation called UniRep,^191^ which was further tuned in an unsupervised manner to obtained eUniRep (evotuned UniRep) for proteins related to the target sequence, leading to an informative set of features for proteins in general as well as for the target in particular. Such representation enabled data-efficient supervised learning of a model for guiding *in silico* evolution.^192^ Alternatively, an unsupervised “probability density model” has been used to produce an “evolutionary density score”, a feature that was then used to augment a small number of labeled data points on which a light model was trained in a supervised manner. Interestingly, such an approach was shown to outperform the supervised **fine-tuning** of the probability density model pretrained in an unsupervised manner.^193^ Another approach^194^ combines self-supervised large protein language models with a supervised structure-to-sequence predictor in a new and more general framework called LM-design that is claimed to advance the state of the art in predicting a protein sequence corresponding to a starting backbone structure, sometimes called “inverse folding”. While inverse folding does not explicitly search the mutational landscape, it can be used to identify promising mutations by inputting an existing protein structure and a partially masked sequence and using the inverse folding tool to propose amino acids for the masked parts.

### 3.4 Leveraging Unlabeled Data Sets to Score Mutations

Over the past decade, **large language models** (LLM) have become popular tools for solving NLP problems ranging from language translation to sentiment analysis.^195^ This major paradigm shift was driven by the realization that even unlabeled data contain useful information: the distributional hypothesis suggests that the meaning of words can be deduced by analyzing how often and with what partner words they appear in various texts.^196^ Analogously, in biology, we can regard proteins as sequences based on “the grammar of the language of life”, which implies that the distribution of amino acids at specific locations can provide valuable insights that could be used to help predict the effects of substitutions on protein function, thereby reducing the reliance on external data sources.^66^ For example, Elnaggar et al. showed that the embeddings generated by a LLM, when used as input features, can effectively facilitate the development of small supervised models whose predictive power rivals that of state-of-the-art methods relying on evolutionary information obtained from MSAs.^197^ Additionally, the protein language model ESM-2 was recently trained on protein sequences from the UniRef database to predict 15% of masked amino acids in a given sequence.^67^ This made it possible to directly leverage sequence information to greatly improve B-cell epitope prediction^198^ without a supplementary MSA. The inherent attention mechanism of ESM-2 can also be used to facilitate protein structure prediction.^67^ We provide more examples in Table 3. Furthermore, the embeddings obtained from the language models, such as ESM-1b,^65^ ESM-2,^67^ ProtT5,^197^ and ProtTrans,^199^ have become a popular way of representing sequential data, making pretrained ESM models a frequent subject for **transfer learning**. The transfer of knowledge from these models has been tackled by fine-tuning the pretrained weights,^197,200^ by training models solely on top of the learned embeddings (keeping the pretrained weights fixed),^197^ and also by introducing adapter modules^201^ between the trained layers for parameter-efficient fine-tuning.^202^ The power of the learned embeddings can also be witnessed in a completely unsupervised setting. More specifically, the ESM-1v^66^ model was demonstrated to accurately score protein variants by relying solely on the wild-type amino acid probabilities learned by the protein language model during pretraining without any subsequent fine-tuning.

**Table 3:** Some Recent Self-Supervised ML Models for Protein Engineering^a^

approach	tool	training data	method	Web site
large sequence-based models	ESM-2 (67)	∼65 million unique sequences sampled from UniRef50 (203)	transformers, 15 billion parameters, masked (15%) language modeling	https://github.com/facebookresearch/esm
	ProtTrans (197)	2122 million sequences from Big Fantastic Database used for pretraining and fine-tuning on 45 million sequences from UniRef50 (203)	transformers, 11 billion parameters, masked (15%) language processing	https://github.com/agemagician/ProtTrans
	Ankh (199)	45 million sequences from UniRef50 (203)	transformer, 1.15 billion parameters, 1-gram span partial demasking/reconstruction (20%)	https://github.com/agemagician/Ankh
	ProGen^35,204^	281 million nonredundant protein sequences from > 19 000 Pfam^205^ families	transformer-based conditional language model, 1.2-billion parameters	https://github.com/salesforce/progen
	Tranception (164)	249 million sequences from UniRef100 (203)	autoregressive transformer architecture, 700 million parameters	https://github.com/OATML-Markslab/Tranception
large structure-based models	MutCompute (31)	19 436 nonredundant structures with resolution better than 2.5 Å drawn from structures in the PDB-REDO database^206^	3D convolutional neural network, amino acid local environment separated into biophysical channels	https://mutcompute.com/
	ProteinMPNN (207)	19 700 single-chain protein structures,^78^ with resolution better than 3.5 Å and <10 000 residues in length	message-passing neural network, 1.6 billion parameters	https://huggingface.co/spaces/simonduerr/ProteinMPNN
				https://github.com/dauparas/ProteinMPNN
	FoldingDiff (27)	24 316 training backbones from the CATH data set^208^	denoising diffusion probabilistic model	https://github.com/microsoft/foldingdiff
	RFdiffusion (28)	structures sampled from PDB^78^	denoising diffusion probabilistic model	https://github.com/RosettaCommons/RFdiffusion
	GearNet (68)	805 000 predicted protein structures from AlphaFoldDB^209^	graph neural network, 42 million parameters	https://github.com/DeepGraphLearning/GearNet
	Stability Oracle (33)	22 759 PDB structures^78^ with resolution better than 3 Å, maximum 50% sequence similarity, and ∼144 000 free energy labeled data points for fine-tuning	graph transformer, 1.2 million parameters	N/A
models for specific protein families	MSA Transformer ^210^	26 million MSAs with 1192 sequences per MSA on average	transformers, 100 million parameters, axial attention (211)	https://github.com/rmrao/msa-transformer
	ProteinGAN (212)	16 706 sequences of bacterial malate dehydrogenase	generative adversarial networks, 60 million parameters	https://github.com/biomatterdesigns/ProteinGAN
hybrid methods	Prot-VAE (213)	46 million sequences from UniRef sequences and ∼5300 SH3 (Src homology 3 family) homologues	transformers, 1D convolutional neural network and a custom architecture	N/A
	ECNet (214)	protein sequences from Pfam^205^	transformers with a 38 million parameter model, bidirectional LSTM^215^	https://github.com/luoyunan/ECNet
	ESM-GearNet (32)	805 000 predicted protein structures from AlphaFoldDB^209^ and ∼65 million unique sequences sampled from UniRef50 (203)	transformers, 15 billion parameters, masked (15%) language modeling (sequences), graph neural network, 42 million parameters (structures)	https://github.com/DeepGraphLearning/GearNet

a N/A = not available

Large deep-learning models are impressively capable of learning general protein properties. For the cases in which a detailed understanding of a specific protein or protein family is required rather than general patterns that hold across different protein families, sequence-based models that can learn distributional patterns from a single MSA are available. MSAs have already proven to be a rich source of evolutionary information, *e.g.*, for identifying functionally important conserved regions, insertions, or deletions to clarify the mechanisms driving sequence divergence.

Analyses of evolutionary data and comparisons of assigned probabilities for mutant and wild-type sequences have also shown that ML-based models can predict the effects of mutations in deep mutational scanning experiments more accurately than established methods based on evolutionary data.^66^ For example, excellent protein structure prediction performance was achieved by combining MSA inputs with an advanced transformer architecture.^99,210,216^ In addition, Xie et al. used the maximum entropy (MaxEnt) principle to infer statistical energy from homologous sequences^143^ and found that the inferred statistical energy of active site residues correlated significantly with enzyme activity, whereas that of residues distant from the active site correlated with protein stability. Hsu et al. observed that a hybrid linear regression model combining the evolutionary density score from MSA-based ML models with labeled one-hot encoded protein sequence data sets demonstrated superior performance in a range of protein fitness prediction tasks, even for the sizes of the labeled data sets in the range of 48–240 data points.^193^ A similar effect for small data sets of 50–250 data points was reported by Illig et al.^217^ Ding et al. showed that variational autoencoders can capture phylogenetic relationships in the geometry of the latent space of an MSA and further demonstrated that the free energies of sequences assigned by the latent model can be used to predict mutation-induced changes in stability.^218^ Building on these findings, the geometric structure of a latent space was recently used to guide the design of a haloalkane dehalogenase.^219^ In addition, by exploring the latent manifold underlying the sequence information, we can uncover dependencies that may not be readily apparent in the raw latent space embeddings.^220^

Despite its advantages, MSAs also have some drawbacks. First, it can be difficult to create an MSA that contains enough evolutionarily relevant sequences to establish strong patterns at key amino acid positions. Second, the creation of an MSA is often seen as something of a craft rather than a systematic procedure, and the alignment process can be sensitive to user choices, including the choice of substitution matrices, gap penalties, and the number of iterations. Poor choices may produce an MSA with improperly aligned residues because too few iterations were performed during the alignment process or because a significant number of gaps were introduced (particularly at the beginning and end of the alignment), necessitating additional preprocessing and trimming of the MSA to achieve optimal results. Moreover, aligning new sequences to an MSA can be a challenging task that requires careful consideration of the alignment parameters and sequence properties. Despite these challenges, MSAs remain a powerful source of information for studying the relationships and evolutionary history of biological sequences.

Recent studies have also explored the opportunities provided by examining patterns at two contrasting scales within the protein sequence space: general patterns in the large universe of protein sequences and local distributions of sequence patterns in specific protein families. These works have combined large pretrained models with lightweight, easily retrainable components for efficient family-specific adaptation. For example, Sevgen et al. designed ProtT-VAE, a fusion of a transformer pretrained on 46 million UniRef50 sequences and an autoencoder that enables fine-tuning on a sequence library of interest.^213^ After fine-tuning the model on the phenylalanine hydroxylase (PAH) sequence family, ProtT-VAE was used to predict variants with up to 100 mutations, resulting in a 2.5× increase in catalytic activity over the human wild-type. Similarly, Luo et al. combined a pretrained language model with an MSA-specific direct coupling method to capture both general protein syntax and protein-specific epistasis.^214^ The resulting model, ECNet, was used to engineer TEM-1 β-lactamase variants with improved ampicillin resistance.

The utilization of unlabeled data in a semisupervised setting has been tried, for example, in the context of secondary structure prediction for orphan sequences^221^ or structural similarity prediction for protein sequences.^222^ These two methods employ pseudolabeling or a custom similarity metric to enable a supervised learning task to profit from large initially unlabeled data. In some cases, even self-supervised methods are viewed as semisupervised^223^ in that they learn powerful representations from unlabeled data that can then be fine-tuned using small labeled data sets. However, the difference from semisupervised methods is that self-supervised methods build on the established methodology of supervised learning as they formulate a supervised proxy task using synthetic labels generated automatically from the large unlabeled data set. Such self-supervised methods are powering the recent successes of protein language models, discussed at the beginning of this section, and appear to be generally more successful than traditional semisupervised methods.

In parallel to the explosive growth of sequence databases, the Protein Data Bank reached the landmark value of 200 000 entries in April of 2023, providing a rich source of experimental structural data for self-supervised learning about protein structures. A number of deep learning models have been fitted to large subsets of the PDB data to leverage the natural diversity of the protein structures. For example, MutCompute was trained on a nonredundant sample of 19K proteins to predict artificially masked residues based on the local 3D environment, enabling the model to successfully capture phenotypic landscapes associated with protein stability.^31^ Additionally, Zhang et al. designed multiple general tasks for the self-supervised pretraining of graph neural networks for protein structures, leading to improved performance in various downstream tasks.^68^ Self-supervised learning on protein structures has also been used to suggest protein sequences for specific backbones and generate *de novo* protein structures.^27,28,207^

### 3.5 Leveraging Protein Dynamics to Compare Mutations

The preceding sections focus on the analysis of static data. However, proteins are complex and dynamic systems, and their conformational changes and motions often provide key insights into reaction mechanisms that cannot be obtained by studying static structures alone.^224^ One way to capture a protein’s dynamics is by studying its structural ensembles, which are available from existing protein structure databases in many cases. It has therefore been suggested that structure-based ML methods trained on such data, *e.g.*, AlphaFold2, could provide insights into protein dynamics.^225^ Building on this hypothesis, Brotzakis and coauthors proposed a reweighting procedure using AlphaFold2 predictions and the FoldingDiff framework^27^ to generate structural ensembles for disordered peptides.^226^ Such procedures could be useful alternatives to computationally expensive simulations in some cases. However, they are unlikely to be useful for comparing structural ensembles of closely related protein variants because AlphaFold2 predictions, which rely on evolutionary information obtained from MSAs, appear to be insensitive to single-point mutations^139^ and so may be unable to accurately capture the often subtle differences between closely related protein variants.

Another option is to study molecular dynamics (MD) trajectories. MD data are obtained by performing simulations that apply physical laws to calculate the future position of every atom in a protein after a given time step based on its 3D structure at the current point in time. The resulting trajectories consist of a series of snapshots capturing the protein configurations at successive points in time. Because each snapshot contains the coordinates of all atoms in the simulated system, the simulation provides a large amount of data, even for an average-sized protein. Where prior system knowledge is available, one can use a coarse-grained model to reduce the system’s dimensionality and potentially capture major motions of interest.^227^ In most cases, however, finding so-called collective variables (CVs) that effectively describe the system’s dynamic behavior is not straightforward. It has been suggested that unsupervised learning methods could help solve the problem of identifying CVs because they can learn from raw MD trajectories without making assumptions about the CVs that are being sought. In general, such methods try to find low-dimensional representations that are rich enough to reconstruct the original (or time-shifted) input.^228^ The resulting low-dimensional projections can then be used to build a simplified model of the protein’s dynamics. This is often done using Markov state models (MSMs), which can cluster the conformational space of the simulated molecule into a tractable number of clusters (states). MSMs assume that the transitions between these states are Markovian, *i.e.*, the state in the next time step depends only on the current state regardless of the previous trajectory. This approach was applied in conjunction with the end-to-end deep learning model VAMPnet, which was used to find an optimal projection for the system under study.^229^ More recently, the CoVAMPnet framework^230^ was created by expanding on the VAMPnet approach to add interpretability capabilities and a method for MSM alignment that facilitates comparisons of MSMs for two sets of simulations. If applied to different variants of a given protein, CoVAMPnet could potentially be used to evaluate the effects of discriminative mutations on protein dynamics. However, this application is yet to be explored.

In general, identifying dynamic features associated with biochemical differences between protein variants is much more challenging than finding a low-dimensional representation for a single protein. One way to address this problem was demonstrated by the DiffNets framework,^231^ in which supervised autoencoders were trained on MD trajectories to identify the most significant differences in pairwise residue distances between protein variants. Another approach for comparing dynamical changes in variant trajectories involves directly analyzing the distribution of low-dimensional projections of configurations obtained by variational autoencoders. In this approach, similar spatial configurations tend to cluster in certain subspaces of the learned low-dimensional space. Such clustering can be used, for example, to analyze the MD simulations of catalytic sites in the presence of different substrates to identify structural differences that drive substrate preferences.^232^ Nonetheless, despite those promising studies, the problem of comparing MD trajectories of different protein variants in a systematic way remains largely unexplored, offering an intriguing future direction for machine learning applications.

## 4 Recent Success Stories and Lessons Learned

While the number of publications on ML for protein engineering is growing rapidly (Figure 1), only a fraction of these publications incorporate the experimental validation of generated predictions as opposed to validation using existing data. This section highlights several recent publications that do include experimental validation and clearly demonstrate the great potential of ML in enzymology. We provide more examples of such studies in Table S1.

### 4.1 Sequence-Based Case Studies

Several publications have showcased recent advances in the use of sequence-based models, including both models trained on large sequence databases and models trained on specific enzyme subfamilies. For example, Russ et al. used a statistical model based on direct coupling analysis to learn the natural distribution of amino acids in chorismate mutases and generate 1618 artificial enzyme sequences. Experimental studies showed that 30% of these proteins rescued the growth of enzyme-deficient *Escherichia coli* in minimal media.^233^ Ten of them were characterized in more detail and shown to have expression and catalytic parameters similar to those of the previously characterized enzymes. In another case, Repecka et al. used generative adversarial networks to create artificial malate dehydrogenases.^212^ Sixty of the resulting artificial proteins (which had pairwise sequence identities of 45–98% with natural enzymes) were tested experimentally, revealing that 13 had catalytic activity comparable to that of natural enzymes. Another interesting case study was reported by Madani et al., who used a large 1.2 billion-parameter language model called ProGen for the conditional generation of 100 artificial sequences that were fine-tuned to five distinct lysozyme families.^35^ Again, the artificial designs had expression levels and catalytic efficiencies similar to those of natural lysozymes even though their sequence identities with the natural proteins were as low as 31.4% in some cases. In all three studies, the authors could approach the expression and activity of natural sequences, but surpassing them by a significant margin still remains a challenge.

### 4.2 Structure-Based Case Studies

Designs superior to wild-type templates could potentially be created by leveraging structural data. One promising strategy for this purpose is to use the local structural environment to identify positions suitable for optimization in wild-type proteins. An insightful recent study on plastic-degrading enzymes demonstrated the power of this approach.^234^ Despite extensive research on enzymatic PET depolymerization, most known PET-hydrolyzing enzymes have poor activity and require high temperatures or highly processed substrates to be practically useful. Traditional protein engineering strategies have improved the thermostability and catalytic activity of PETase variants under certain conditions, but these variants still show low activity at mild temperatures. Lu et al. therefore tried to use structure-based deep learning to solve this problem.^234^ For this purpose, they used the MutCompute^31^ algorithm, which was trained to predict masked amino acids based on their local 3D microenvironment in a crystal structure, to identify the positions where the predicted probabilities of the wild-type amino acids were comparatively low, suggesting that some other amino acids are better “suited” to the corresponding structural microenvironment. Eight of the top ten suggested substitutions produced single-point mutants with improved thermostability and activity. Combinatorial assembly of these substitutions yielded PETase variants with melting temperatures of up to 83.5 °C (and thus greater thermal stability than any previously reported variant of this enzyme) and up to 38× the activity of the template enzymes at 50 °C.

MutCompute was also used to improve the thermostability of the Bst DNA polymerase, with a similar success rate: of the top 10 substitutions suggested by the network, only two were discarded for showing little or no activity, while the top five yielded activities equal or superior to the template.^235^ Moreover, variants combining these substitutions were more robust to purification and exhibited even greater thermotolerance and thermostability. MutCompute predictions are orthogonal to force-field calculations and phylogenetic analyses conducted with the popular automated web tools PROSS^236^ and FireProt,^237^ and these predictions can be exploited to remove destabilizing substitutions from multiple-point mutants designed using those tools. This was recently demonstrated by the successful stabilization of haloalkane dehalogenase DhaA115 (PDB ID 6SP5(238)).

An alternative to replacing specific residues to better match structural patterns in training data is to predict complete protein sequences corresponding to a given backbone conformation from scratch. Exemplifying this approach, Dauparas et al. trained the graph-based neural network ProteinMPNN on a set of 19 700 high-resolution single-chain structures from PDB and showed that this algorithm can rescue previously failed designs by suggesting optimized protein sequences for given scaffold templates.^207^ For experimental validation, the authors targeted proteins that had been generated by deep network hallucination in a previous study^239^ but proved to be mostly insoluble when expressed in *E. coli*. Ninety-six designs were processed using ProteinMPNN, of which 73 were soluble when expressed and 50 had the desired monomeric or oligomeric state. Moreover, some maintained this state even at 95 °C. This is a promising result because protein insolubility appears to be a persistent problem with ML-based generated sequences.^240^ It will therefore be interesting to see if more enzyme sequences can be reengineered for improved solubility in this way.

At Loschmidt Laboratories, we developed the sequence-based solubility predictor SoluProt^26^ and integrated it into EnzymeMiner (https://loschmidt.chemi.muni.cz/enzymeminer/), a more general pipeline for discovering enzymes with a desired catalytic activity that is available as a fully automated web service. This pipeline was recently used to mine promising industrially relevant haloalkane dehalogenases^241^ and fluorinases.^242^ In both cases, we obtained soluble and active enzymes with catalytic performance superior to that of previously characterized wild-type enzymes and engineered biocatalysts. The broad applicability of the SoluProt and EnzymeMiner web services is demonstrated by the fact that they have completed over 30 000 and 3000 jobs, respectively, in the two years that they have been online. An alternative to natural sequence mining with EnzymeMiner is to use the deep neural network ProteinMPNN, which has been applied successfully in the *de novo* design of artificial luciferases based on computationally designed binding pockets.^243^ Functional constraints have also recently been incorporated into structure-based generative models including diffusion-based deep learning methods for functional motif scaffolding.^28^

### 4.3 Small-Data-Set-Based Case Studies

The preceding examples demonstrate the potential of using unlabeled data sets to suggest novel protein designs. Moreover, in section 3 we discussed strategies that leverage small data sets, for example, to fine-tune pretrained large models in order to enable more focused protein engineering. However, ML models trained using only small data sets with no pretraining can also be useful in protein engineering pipelines using simpler algorithms.

Several case studies combining machine learning and directed evolution have appeared recently. Based on the initial *in silico* docking, Büchler et al. chose three critical amino acid positions in an iron/α-ketoglutarate-dependent halogenase for full randomization in a library targeted for the use in an algorithm-aided enzyme engineering strategy.^244^ After collecting the activity measurements for 504 unique variants, the authors trained a Gaussian-process-based model to explore *in silico* the remaining protein landscape for activity and selectivity. The subsequent experimental validation revealed active and selective halogenase variants with over a 90-fold increase in the apparent *k*_cat_ and a 300-fold increase in the total turnover number. A smaller data set of 80 variants of Sortase A was used by Saito et al. to train an ML model to score all possible variants for five mutated positions.^245^ After designing primer sequences to include the top 50 variants in the second-round library, the authors observed most of the new variants showed high expression levels, with several demonstrating higher enzyme activity than the first-round variants. Reiterating the workflow, the authors constructed and validated the third-round library, again leading to a set of improved variants. Interestingly, the authors tested and stressed the importance of including poor-performing variants in the training data, and they still got promising results in the scenario in which the top-performing variant was excluded from training. Even a smaller initial library of 20 chimera sequences was used in a different study by Greenhalgh et al. as a starting point for ten rounds of sequence optimization of alcohol-forming fatty acyl reductases, leading to an over twofold increase in fatty alcohol production compared to the starting sequences.^246^

In all three case studies above, the authors used a set of simple sequence-based features such as one-hot encoding of amino acids, physicochemical properties of the proteins, or conservation scores. While extracting features from protein dynamics remains challenging (see section 5.2), a linear model was recently used to this end to elucidate structure–function relationships while engineering luciferases.^247^ This study drew on an earlier indel (insertions and deletions) mutagenesis experiment targeting a reconstructed ancestral protein and aimed to identify the factors responsible for the emergence of dual dehalogenase and luciferase activities.^248^ The authors comprehensively studied the dynamics of different variants and used partial least-squares regression to identify the strongest predictors of both activities. This knowledge was then used to obtain a design with lower product inhibition and highly stable glow-type bioluminescence.

These examples are notable because while few groups have the expertise or infrastructure needed for deep learning, simpler and more accessible ML methods can still be used to advance traditional protein engineering pipelines. At the same time, we expect that deep learning tools will gradually become more accessible and easier to use.

## 5 Major Gaps in the State of the Art

Despite the exciting applications and promising case studies discussed above, several significant knowledge gaps remain to be addressed to take protein engineering to the next level. First, many protein engineering tasks have yet to benefit from ML, including predicting the effects of indels and unnatural amino acids, creating predictors that address several targets simultaneously, and predicting mutational effects on protein interactions. Second, molecular dynamics remains isolated from major advances in the application of ML to protein engineering; dynamical information is rarely if ever used when training current state-of-the-art predictors. Third, there is a pressing need to establish gold standard protein data sets because such benchmarks have significantly accelerated the progress of ML in other domains. Finally, the impact of ML-based tools often remains limited to a narrow circle of method developers, so there is a need to reach out to the broader community of biochemists and synthetic biologists. Below we discuss each of those gaps in more detail.

### 5.1 Unexplored Protein Engineering Tasks

One important objective for the future will be to create single ML tools that can perform multiple tasks. Multiobjective protein engineering is a core goal of many ongoing experimental studies because the introduction of mutations targeting one property often affects others. For example, stability and solubility are often negatively correlated and may also need to be traded off with other properties such as catalytic activity.^249^ However, current ML-based predictors typically target only one property at a time. This limitation could potentially be overcome by combining predictions from multiple tools to define a so-called Pareto front, *i.e.*, a set of solutions in which no one member is better than another with respect to all objectives.^250,251^ However, by combining the separately trained predictors, one misses the opportunity to train on a larger pool of data sets and potentially capture the common underlying mechanisms in a unified protocol. Approaches of this type are rarely used, possibly because their implementation would require expert knowledge of multiple types of data and the experimental techniques used to generate them.

Another major goal is predicting the effects of insertions and deletions in the amino acid sequence (indels). This area has been largely unexplored by ML even though indels occur frequently in nature and can unlock unique functional changes that substitutions alone cannot achieve.^252^ Indel engineering is gaining momentum, however, and experimental studies focusing on indel mutagenesis are starting to produce interesting labeled data sets.^253,254^ Protein evolutionary information is another potential source of data on indels^255^ that can be used to explore previously hidden catalytic activities that could be shifted or promoted.^256,257^ Indels can affect not only the structure of the protein system but also its dynamics.^258^ Therefore, molecular dynamics simulations may provide valuable data for clarifying the effects of indels on protein dynamics and their functional consequences.

The vastness of the combinatorial protein sequence space can be further extended by introducing unnatural amino acid (UAA) substitutions. Experimental studies have successfully incorporated over 150 different UAA substitutions into protein sequences,^259^ including multiple point mutants.^260^ These substitutions can be used for diverse purposes, ranging from tailoring the structural, physical, and dynamic properties of specific sites to introducing new properties by adding carefully designed UAAs. Such additions can be used to enhance enzyme activity or elucidate the enzyme reaction mechanisms.^261^ While there are some emerging ML-based tools for rational design of UAA sites,^262^ further research and development are needed to make this approach widely applicable in enzyme engineering.

Another area that would benefit from the greater use of ML is the design of protein–protein and protein–ligand complexes. Despite recent promising results in the use of ML to predict the binding sites and protein–ligand complexes,^29,263,264^ reliable and practically useful approaches for designing noncovalent interactions are still missing. For example, Geng et al. found that the evolution of ΔΔG predictors for protein–protein interactions had been hindered by the absence of centralized benchmarking.^265^ Little progress has been made in this area, as evidenced by the re-emergence of similar ML models and the persisting lack of common evaluation standards.^156,266,267^ Furthermore, the reliability of existing models is undermined by a frequent reliance on supervised ML with limited annotated data.^268^

Finally, most ML-based predictors represent primary protein sequences at the level of amino acids. However, synonymous mutations that do not change the amino acid sequence can still significantly impact protein expression and function^269,270^ or can even relate to particular structural features.^271,272^ Predictors working on the level of nucleotides or codons may thus be better tools for protein design and modification, particularly in areas such as prediction of expression, solubility, and aggregation. The fact that the 64-letter codon alphabet serves to encode richer information than the 20-letter amino acid alphabet can be directly exploited by ML models for improving performance on a wide range of tasks that are now being tackled at the protein sequence level.^273^ While there have been several studies, *e.g.*, tackling protein expression optimization,^274^ melting temperatures, subcellular localization, solubility or function,^273^ we believe further research in this area might provide a strong boost for predicting many essential protein characteristics but will require rethinking of the existing data sets at the nucleotide level.

### 5.2 Learning from Protein Dynamics

Protein dynamics profoundly influence biological phenomena and properties ranging from enzyme mechanisms to protein stability and must therefore be taken into account when designing enzymes.^275^ The value of analyzing protein ensembles rather than single-protein structures has been demonstrated in contexts including predicting thermal stability^276^ and identifying the long-range conformational dynamic effect on residues involved in substrate reorientation^277^ and reactivity-promoting regions.^278^ Therefore, tools that can generate dynamic data without extensive computational or experimental data collection would be extremely valuable. This has motivated the development of new methods for generating representative ensembles from structure^279^ or sequence data.^280^

Despite the growing availability of protein dynamics data from sources such as MD simulations, current methods for predicting protein properties are mainly based on single sequences or static structures. This is partly because several challenges must be overcome when MD results are incorporated into the training and inference phases of classical ML pipelines. The first challenge concerns data representation: predictive models typically work with static structures. One possible strategy could be to include a limited set of quantities derived from a trajectory, *e.g.*, the flexibility of structural elements expressed in terms of their B-factors, in the set of input features. Another possibility would be to select a subset of conformations to be used during training. For example, one might use apo and holo structures or representative conformations chosen with various clustering methods or Markov State Modeling. However, the applicability of these methods to training data sets of multiple proteins has yet to be explored.

An interesting recent approach that could help with data processing is “dataset distillation”,^281^ which builds on research related to distillation of neural network models.^282^ In data set distillation, the goal is to distill a larger data set into a smaller one of potentially artificial examples so that models trained on the smaller data set can match the performance of those trained on the larger one. This process was first demonstrated in the image recognition domain by distilling the well-known MNIST data set^283^ of 60k images into just 10 synthetic images (one per class); models trained on the distilled data set achieved performance closely approaching that of models trained on the original data set.^281^ At present, data set distillation has been most beneficial for models that are retrained multiple times, such as those used in **continual learning** or NN architecture searching. The use of data set distillation is conditioned on the existence of large data sets and relevant models that can be trained on those data. So far, relatively little work has been done on distilling temporal data such as videos,^284^ but as soon as data set distillation demonstrates its utility in the domain of video analysis, we expect this technology to become applicable to processing of MD trajectories as well. Data set distillation techniques have recently been comprehensively reviewed by Yu et al.^285^ and Lei et al.^286^

The lack of training data and benchmarks appears to be another fundamental barrier to the systematic integration of MD data into ML-based protein engineering pipelines. Compiling data sets of MD trajectories presents both technical and scientific challenges resulting from the inconsistent file formats due to the use of different simulation packages,^287^ huge file sizes, and the fact that MD data are rarely published. Furthermore, MD trajectories can be very sensitive to the choice of force fields^288^ and other settings. This high variability of simulation data makes compiling large, consistent, high-quality data sets truly challenging. The application of the FAIR principles^289^ to the publication of biomolecular simulation results is thus an important first step toward building relevant data sets and benchmarks and will encourage further development of the field by greatly increasing data availability. The initiative of publishing the MD trajectories is also called upon by Tiemann et al., who performed an extensive MD data mining exercise and demonstrated the utility of publicly accessible data.^290^ The prioritization of building data sets for specific proteins or protein families appears to be a reasonable next step, followed by exploring the possibility of transferring knowledge between different protein families.^291^

### 5.3 Missing Gold-Standard Data Sets

High-quality data benchmarks are major drivers of progress in ML research.^292^ For instance, the remarkable progress in image classification can largely be attributed to the existence of well-prepared and maintained benchmarks: an early example was the MNIST collection of hand-written digits,^283,293^ which was later complemented by the PASCAL visual object classification (VOC) challenge data set of real photographs^293^ and the ImageNet data set.^294^ MNIST enabled the first successes of deep learning on hand-written digits,^295^ while the PASCAL VOC data set and the associated challenge established standard benchmarking practices in computer vision research, including the use of a hidden test set. Over the past decade, the benchmarking of progressively more advanced convolutional neural networks on ImageNet has produced models with superhuman classification performance.^296^ Here it is important to stress the difference between a data set and a benchmark data set: to qualify as a benchmark, a data set must satisfy stringent quality criteria, have well-defined benchmarking tasks and performance metrics, and have a predefined split into training and test sets.^297^ Moreover, the performance of all models on the MNIST data set is measured in terms of the percentage of wrong classifications. Such universally accepted benchmarking criteria enable clear and fair evaluations of the practical performance of proposed models, eliminate the need to spend time on data preparation, and introduce an element of competition into model development.

While such benchmarking practices are standard in traditional ML domains, they remain far from common in protein engineering for several reasons. First, the complexity of the domain presents challenges in collecting data and ensuring their quality. The quality of ImageNet was guaranteed by manual verification of each image, which was achieved by creating a special interactive web site to which any nonexpert could contribute. The bulk of the annotation of one million images was done using annotation services, such as Amazon Mechanical Turk. However, protein data require much more involved manual curation by domain experts, making such approaches unusable. Automated curation may be feasible for some types of data, *e.g.*, MMseqs2 and Foldseek enable fast deduplication and clustering of sequences^80,298^ and monomeric structures.^80,298^ However, it is extremely difficult for data sets focusing on the catalytic activity, specificity, enantioselectivity, or solubility. Large-scale data cleaning also remains challenging for protein–protein interfaces, which are highly repetitive due to the redundancy of the PDB.^31,299,300^

Next, the selection of a suitable ML evaluation metric and data split may be challenging because of the inherent interdisciplinarity of protein engineering. The establishment of benchmark components requires a deep understanding of the intricacies of biochemistry as well as expertise in ML. For example, mutational data sets can be split at the level of specific substitutions, mutation sites, specific proteins, or protein families while ensuring that no entry in the test set is repeated in the training data. However, even in a simple setting using protein sequences, splitting at the protein level becomes challenging if the data set contains many homologs or protein variants, and many authors use different sequence identity cutoffs for clustering data before splitting. Protein structure-based learning introduces additional complexity into the process of defining splits. Distinct sequences may have very similar tertiary structures, necessitating the use of more advanced geometric or graph-based splitting conditions. However, such methods are rarely applied, leading to the use of a wide variety of splits that limits model comparability.^156^ For example, a widely used data set that was reported to suffer from improper splitting^301^ is PDBBind.^302^ Simple random splits may result in data leakage, especially when using highly redundant or nonuniform data sets.^77,303,304^

The Critical Assessment of Structure Prediction (CASP) is the best example of a well-established benchmark in protein science.^305^ It has been produced and maintained by a community-driven effort that has been ongoing for almost three decades, enabled the success of AlphaFold2, and is continually being extended to new and more complex tasks.^99^ Despite the existence of several other CASP-inspired benchmarks such as Critical Assessment of Prediction of Interactions (CAPRI) and Critical Assessment of Function Annotation (CAFA),^306,307^ standard benchmarks for protein design are still lacking. This problem is attracting attention; for example, Dallago et al. recently introduced the Fitness Landscape Inference for Proteins (FLIP) benchmark,^303^ which addresses three protein engineering problems by including: (i) an almost complete mutational landscape (149 361 of 160 000 variants) of four strongly epistatic residues of the G protein, (ii) the more diverse and sparsely sampled landscape of the AAV capsid protein, and (iii) the highly diverse thermostability landscape of proteins from different domains of life. Similar benchmarks are also appearing in related disciplines such as genomics^308^ and drug discovery.^75,309^ Calls for defining clear criteria for the study of epistasis and residue coevolution patterns can be found in the literature;^175^ these criteria could enable the emergence of new benchmarks. We hope to see more initiatives creating and supporting benchmarking in the future, as they could dramatically accelerate progress in ML for protein design.

### 5.4 Poor Transfer of Knowledge from Concept to Application

Even when authors have performed appropriate independent method validation and convinced readers of the superiority of their tool, the transfer of knowledge from published methodologies to new applications seems to be frustratingly slow. Despite the rapidly growing number of publications describing applications of ML in protein engineering, surprisingly few studies have followed up on published methods and applied them to new protein targets. The main obstacle to such work appears to be the limited accessibility of ML methods to researchers without expertise in computer science, *e.g.*, biochemists. One established scientific publishing standard for new ML methods is that protocols and scripts should be included with the submitted manuscript.^310^ While this improves the peer-review process, potential future users of such tools are generally less comfortable running such scripts than reviewers. Therefore, if a new method is available only as a collection of scripts in a GitHub repository, it is unlikely to have much impact outside the narrow community of ML developers.

Creating at least a minimalistic user-friendly interface is thus vital for making methods widely accessible to users other than their developers, even though it generally requires additional work from the developers that is outside the scope of their research objectives. The explosive growth in the number of users of ChatGPT 3.5 and later versions has been partially attributed to its simple dialogue-like interface, which is accessible even to users without knowledge of ML. Similarly, the AlphaFold2 release was followed by the dissemination of a Google Colab notebook,^311^ allowing it to be used by researchers in a way that is much more user-friendly than downloading code from GitHub. In Loschmidt Laboratories, we have been developing user-friendly tools for over two decades (https://loschmidt.chemi.muni.cz/portal/), and we often hear how crucial the ease of use of these tools is to our users. We ensure this ease of use by making efforts to support popular input formats (ideally, using file types that a typical user will have at hand), creating intuitive and well-guided settings (ideally, with a default setup that will provide reasonable results in most use cases), and providing comprehensive and easily understood reporting of results. We also strongly encourage other developers to invest time into making their tools accessible to other communities, such as enzymologists or biochemists. Moreover, it is usually helpful to contact past or potential users to understand how they see the method and what functionality they would benefit from in addition to the method itself. This could include fetching sequences and structures from databases, submitting multiple sequences or mutations as a single input, suggesting designs for experimental validation, or even allowing output graphics to be easily transferred to a publication.

We also encourage authors to be more open about the limitations of their tools in publications. When published case studies use additional steps, *e.g.*, manual fine-tuning, these should be discussed explicitly in the main text of a publication or in instructions for using tools. While there is pressure to present a tool’s performance in the most favorable light, any cherrypicking will eventually disappoint future users and undermine the trust in the entire domain. To be successful, ML requires users and the wider community to trust its predictions and be convinced that the tools have been assessed fairly. Consequently, there are active efforts to improve reporting standards for ML tools in modern biology, and several clear guidelines have recently appeared to guide authors in preparing manuscripts dealing with ML.^12,310,312^ However, there will be a lag phase before such rules are universally adopted.

## 6 Future Opportunities

### 6.1 Trustworthiness and Explainable AI

In many domains, including medicine and finance, it is vital for ML systems to be interpretable and explainable to build trust in the algorithms. Understanding the mechanisms behind making a prediction can also enable more rigorous verification of a tool or provide clues for follow-up decision-making. In this context, an explainable model is one that provides insights into the reasons for its predictions and decisions, for example, by showing which parts of the input have the greatest impact on predictions. Conversely, an interpretable model is one for which the internal process of making predictions can be readily understood by humans. This can be achieved by having an explicit decision pathway in a decision tree or easy-to-grasp feature weights in a simple linear equation. If ML algorithms are not mathematically complex, they are often intrinsically explicable and interpretable. However, interpreting and explaining their predictions becomes more challenging as the algorithms become more complicated. This is why models such as deep neural networks are often described as black boxes. The field of explainable artificial intelligence (XAI) seeks to overcome this challenge by creating explanations using analytics, saliency maps, or words to allow humans to understand why an ML algorithm makes a certain decision or prediction.

**Figure 4:**
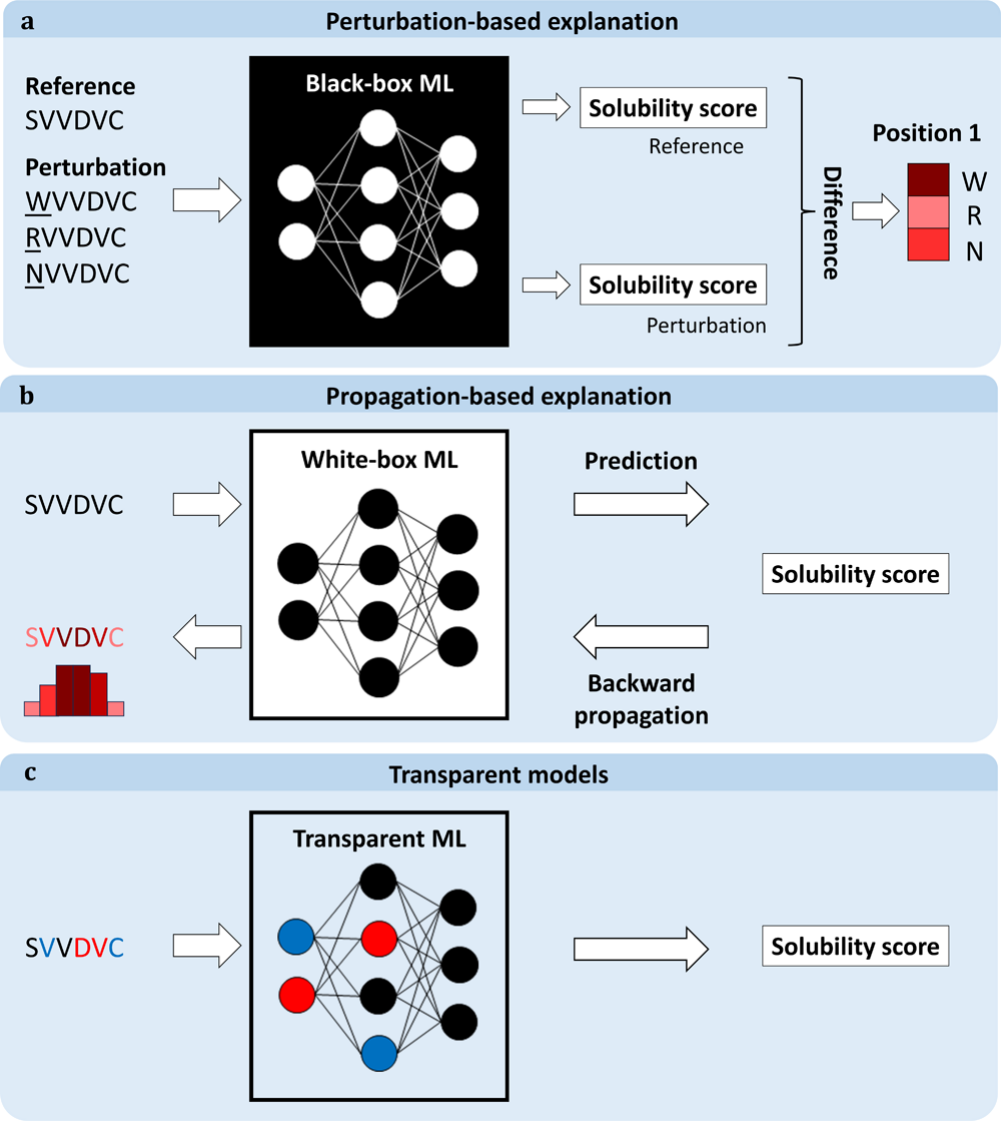
An overview of explainable AI methods. The figure illustrates the concepts using the task of predicting protein solubility from primary structure data as an example. (a) Perturbation methods check the effect of changes in input data on output; a significant change in output causes the corresponding input to be marked as crucial for prediction (darker red colors on the output indicate a higher difference in prediction and hence higher importance for the output). Perturbed amino acids in the input are underlined. (b) Propagation methods use the network structure and move from the prediction to the input to determine which parts of the input have the greatest impact on the prediction (here, darker colors indicate greater impact on the prediction). (c) Transparent networks are designed to be interpretable, for example, by specific choices of their architecture or the individual building blocks (*e.g.*, filters in a convolutional network might be interpreted as specific relevant sequence motifs identified during training). Here different parts of the network (shown in different colors) are related to different parts of the input sequence (illustrated in the same colors).

A range of XAI methods have been proposed in recent years^313,314^ (Figure 4) and are discussed in more detail in two recent comprehensive reviews.^313,315^ Two simple strategies for XAI are to use self-explainable white-box methods and to check how changing inputs affect the outputs of black-box networks. Feature importance methods are a notable class of white-box methods that achieve explainability by identifying essential features based on model parameters such as weights and coefficients. For example, in a linear regression algorithm, scientists can determine the importance of each feature based on the magnitude of the associated coefficients. Propagation-based approaches are another class of white-box methods that are often used for similar purposes in deep learning. One of the recent methods in this class is layer-wise relevance propagation, which propagates predictions from the output to the input using propagation rules evaluated at each node of a neural network.^316^ As this method relies on simple formulas, it does not require computationally demanding sampling and is relatively robust to noise and other artifacts during training.^313^

For black-box models, explainability and interpretability are generally achieved by analyzing the input-output behavior. For example, Shapley values estimate the contribution of each feature by evaluating its marginal impact on the output. Unfortunately, this approach requires significant computational resources and scales poorly with increasing numbers of features.^317^ Another strategy is to approximate a black-box model with a more interpretable analogue. This approach is exemplified by the LIME method,^318^ which mimics the predictions of any classifier in an interpretable and faithful manner by locally approximating the decision boundary using a linear regression or a decision tree. Another black-box method explains model predictions by perturbing the input data and analyzing the differences between the actual output and the output for the perturbed data set. If a particular perturbation of the input produces large differences in output, the changed parts of the data set are marked as being essential for prediction.^319^

Another approach to incorporating explainability into ML pipeline is to pursue explainability during the development of the pipeline rather than when the predictor is released, *e.g.*, by using transparent neural networks.^320^ Such neural networks are designed to be more interpretable and understandable by humans. Prior knowledge, such as existing biological knowledge and experimental data, can help scientists develop such models. For example, a convolutional neural network could be designed to learn filters that correspond to known protein structural motifs.^321^ Alternatively, one could use knowledge-primed neural networks in which nodes correspond to proteins that are connected based on prior knowledge.^322^

The ML methods that are increasingly being applied in protein engineering are often complex and challenging to interpret. Because wet-lab validation of ML-designed proteins and enzymes is expensive and time-consuming, it is vital to reinforce the credibility of AI-assisted protein engineering and ensure that experimentalists can be confident that the methods will produce designs with a good chance of success in the lab. XAI can strengthen credibility by providing insights into the decision-making processes of ML models in protein engineering.^323^ It can also help scientists to improve their algorithms by revealing mistakes and biases.^324^ The use of XAI to explain deep learning networks has therefore recently attracted interest in areas of drug discovery, chemistry, and protein engineering including active ligand searching,^325^ prediction of enzyme EC numbers,^326^ and identifying residues that indicate transitions between active and inactive states in GPCR receptors.^327^

In protein engineering, explainable AI is primarily applied for the analysis of predictions from ML models with the aim of obtaining novel biochemistry knowledge. Namely, Tan et al. introduced ExplainableFold,^328^ a concept designed to improve the understanding of deep learning-based protein structure prediction models such as AlphaFold by residue deletions and residue substitutions. Essentially, the core objective of ExplainableFold is to uncover influential residues responsible for maintaining or altering a folded protein structure. More generally, it was also proposed that the application of tools such as exBERT,^329^ originally designed for visualizing internal representations of transformers, could be employed in protein-trained transformers to highlight relationships among amino acids.^330^ Ultimately, the use of explainable AI for protein design is still in its early phase and we have yet to see its main applications in protein engineering pipelines.

### 6.2 Identification of Hidden Biases

ML models can be biased by the data used in their training, which can reduce their overall accuracy or cause their performance to vary substantially across the data input space, *e.g.*, the protein sequence space or the space of protein structures. In supervised learning, special attention must be paid to the problem of class imbalance,^331−333^ which occurs when there are more training examples for some classes than for others. In such cases, the model might overpredict the major classes.^334^ In unsupervised learning, these biases might be harder to spot and quantify. For example, in natural language processing, large self-supervised language models are typically trained on vast amounts of text that are often sourced from the Internet without thorough data curation. If left unchecked, these models can reportedly generate unjust or oppressive language, which promotes discrimination, exclusion, and toxicity.^335^ The risk of compromising model performance by using biased training data is as relevant in enzyme engineering as in any other field.^31^ However, in contrast to natural languages, it is not directly apparent what adverse biases could be adopted by models in enzymology, as there is no quick and simple strategy to directly validate outputs. Therefore, responsibility and caution are advised, especially when different sources of data are combined, *e.g.*, with the data from human genomic databases, which were reported to be racially biased.^336^

A typical example of a bias in enzymatic data arises in the task of predicting mutational stability, where a predictor given an original and mutated sequence as inputs should report a single number corresponding to the predicted ΔΔG. In general, a random mutation of a natural protein is more likely to be destabilizing than stabilizing,^337^ and this bias often propagates to stability data sets, leading to an issue similar to the class imbalance problem resulting in the overprediction of destabilizing mutations. To combat such effects, it has become standard to exploit the antisymmetry of mutational stability, which arises from the physical principle described by the following equation:^338^ ΔΔ*G*(WT → Mut) = −ΔΔ*G*(Mut → WT). That is, the change in Δ*G* upon mutating a residue is equal to the negative change in Δ*G* that would be induced by the hypothetical inverse mutation. This property makes it possible to augment the training data set by adding artificial inverse mutations from the mutant sequence (Mut) to the wild-type sequence (WT) to obtain a balanced data set. Antisymmetry can also be incorporated by design, ensuring that the property is enforced even when using an imbalanced training set.^339^ Interestingly, efforts to solve the problem of predicting protein stability changes upon mutation have driven progress in other sensitivity studies, *e.g.*, the use of reduced amino acid alphabets to account for bias in the representation of mutations in the training data,^131^ structural sensitivity,^340^ or the “scaffold bias” of using crystallographic structures instead of AlphaFold models in ensembles.^276^ Another task where data set biases were reported is structure-based virtual screening. This problem was recently tackled using an ML-based scoring function in which the authors took care to ensure that feature importance was consistent with human knowledge; this forced the model to learn relevant features regardless of the present biases, leading to better generalizability.^341^

In many cases, however, even a detailed understanding of the studied task will not reveal straightforward paths for uncovering and fixing biases because problems often arise from intricate interdependencies between data points that researchers are unaware of. To combat such issues, the concept of multicalibration has been proposed.^342^ The aim of multicalibration is to make predictors perform more uniformly across different data subclasses. However, the complexity of the proposed approach is linear with respect to the number of possible subsets of the training data and, thus, exponential with respect to the number of training samples. To alleviate this high computational cost, other studies proposed low-degree multicalibration by drawing on the multicalibration and multi-accuracy approaches.^343,344^ These concepts are currently mainly being studied within the domain of algorithmic fairness,^345^ which is largely concerned with ML-based predictors that process personal data on human individuals. It is interesting to see if these concepts will be useful in the context of enzymology, for example, by ensuring that learned predictors have similar performance for different protein families.

### 6.3 Other Promising Methods

One current trend in machine learning is that models are increasing in size in parallel with the increasing amount of data on which they are trained. Models such as ChatGPT and DALL-E^38^ are shining examples of this trend. To increase the size of the data sets available for model training in protein engineering, it may be necessary to use as much available experimental data as possible, accepting that experimental data sets will inevitably vary widely in quality and size. To bridge the gap between small high-quality and large low-quality data sets, meta-learning appears to be a promising strategy. In such an approach, nested optimization could be employed for training on a large set of noisy examples in the inner loop and a small set of trusted examples in the outer loop, thus suppressing the impact of the noise in the larger data set.^346^

Molecular simulations using MD and quantum mechanical methods could be another valuable source of data for training large, data-hungry models. However, these simulations are very computationally demanding, as demonstrated by Musaelian et al., who recently used machine-learned potentials in simulations of biomolecules using 5120 A100 GPUs in parallel.^347^ The hardware requirements of MD using such large data sets could be met using purpose-built supercomputers such as Anton 3.^348^ Advances in quantum computing (QC) technology could also make large-scale molecular simulations more accessible, as well as benefit generative ML tasks^349^ and improve the generalizability of models trained on limited data.^350^ However, the greatest benefit of QC in enzyme engineering will likely result from its expected ability to greatly accelerate quantum and molecular mechanics simulations.^351,352^

Given the trend toward ever-larger models, it is important to consider the cost of their training and the CO_2_ emissions resulting from the training and inference process.^353^ This issue can be addressed by adopting energy-saving ML practices,^354^ implementing architectural redundancy to bypass lengthy training processes,^355^ or obviating the need for large model training by narrowing problems down to simple linear combinations of system-relevant physical properties.^356^ Another important recent trend in machine learning involves lightweight adaptation of large pretrained models to new tasks using adaptor layers.^201,357^ These methods change only a small fraction of the parameters of the large scale pretrained model for the new task, leaving the vast majority untouched. This allows large models to be adapted to new tasks at a fraction of their initial training costs; the time required for adaptation may be as little as several hours on a single eight-GPU machine,^358,359^ eliminating the need for the supercomputer infrastructure used to train the original model. This significantly accelerates model adaptation and makes such training and related research accessible to a wide community of researchers who may lack supercomputer access.

Another promising area of application for ML is further automation of enzyme engineering pipelines, for example, by using **reinforcement learning** (RL) to select structural or biophysical motifs that are important for a target property.^360^ Several recent advances in RL have focused on small molecule design.^361^ However, when combined with recent advances in protein science, RL has also shown promise in the design of protein complexes^362^ or peptide binders with high affinities.^363^ Based on this expansion of its applicability domains, we believe that RL could be similarly useful in enzyme engineering. Enabling more user-friendly pipeline interaction, for example, by creating ChatGPT-like interfaces to simplify the control and running of experiments, can potentially further contribute to the automation of the workflows.

## 7 Conclusions

We live in exciting times that provide many opportunities to explore new horizons in enzyme engineering and machine learning. Many ambitious tasks are already being tackled by cutting-edge data-driven algorithms and approaches, often inspired by their spectacular performance in other domains. The tools created on their basis are already playing integral roles in studies aiming to discover and improve biocatalysts. On the other hand, many more goals are still waiting for the appearance of suitable data sets and data processing protocols to be eventually leveraged by machine learning. There are many challenges ahead, including the creation of reliable and user-friendly tools for generating promising protein designs by users with limited ML knowledge, addressing multiple tasks in one pipeline, incorporating protein dynamics in ML pipelines, understanding the effects of insertions, deletions, or unnatural amino acids, increasing the interpretability of the models, and revealing hidden data biases. One of the most significant takeaways from the success of machine learning in other domains, such as natural language processing or computer vision, is the role of large-scale data collection and curation. As biochemistry data tend to be more challenging to acquire than, for example, images or text, developing mechanisms for open sharing and aggregation of data sets across the entire scientific community could have game-changing effects. Building and maintaining community-wide training data sets and benchmarks, including such ambitious data types as protein dynamics, could create entirely new ways for designing proteins that would shape the future of biotechnology.

## Supporting Information Available

Selected recent case studies combining machine learning and protein engineering and a list of databases and data sets for machine learning applications in protein engineering (PDF)
