# Supplementary material for: Machine Learning-Guided Protein Engineering
Source: ACS Catal. 2023 Oct 13;13(21):13863–95. doi: 10.1021/acscatal.3c02743 (PMC10629210; doi:10.1021/acscatal.3c02743)
Supplement: Supplementary file 1 — cs3c02743_si_001.pdf [file cs3c02743_si_001.pdf]

Supporting Information

Machine Learning-Guided Protein Engineering

Petr Kouba[1,3,4,#], Pavel Kohout[1,2,#], Faraneh Haddadi[1,2,#], Anton Bushuiev[3], Raman Samusevich[3,5], Jiri Sedlar[3], Jiri Damborsky[1,2], Tomas Pluskal[5,*], Josef Sivic[3*], Stanislav Mazurenko[1,2,*]

[1] *Loschmidt Laboratories, Department of Experimental Biology and RECETOX, Faculty of Science, Masaryk University, Kamenice 5, 625 00 Brno, Czech Republic*
[2] *International Clinical Research Center, St. Anne's University Hospital Brno, Pekarska 53, 656 91 Brno, Czech Republic*
[3]*Czech Institute of Informatics, Robotics and Cybernetics, Czech Technical University in Prague, Jugoslavskych partyzanu 1580/3, 160 00 Prague 6, Czech Republic*
[4]*Faculty of Electrical Engineering, Czech Technical University in Prague, Technicka 2, 166 27 Prague 6, Czech Republic*
[5]*Institute of Organic Chemistry and Biochemistry of the Czech Academy of Sciences, Flemingovo nám. 2, 160 00 Prague 6, Czech Republic*
[#]These authors contributed equally to this study.

*Corresponding authors:

Tomas Pluskal (tomas.pluskal@uochb.cas.cz)

Josef Sivic (Josef.Sivic@cvut.cz)

Stanislav Mazurenko (mazurenko@mail.muni.cz)

Table S1. Selected recent case studies combining machine learning and protein engineering. The selection includes examples of ML-based protein engineering campaigns with experimental validation. The initial list was obtained by querying Scopus with the key words “machine learning” and “protein engineering” and restricting the search to articles published after 2020.

**Year of publ.**	**Target protein**	**Property**	**ML method**	**Ref.**
2020	chorismate mutase	activity	Potts model bmDCA	(1)
2021	malate dehydrogenases	activity	generative adversarial network ProteinGAN	(2)
2023	lysozyme	activity	large language model ProGen	(3)
2022	PETase	stability	self-supervised 3D convolutional neural network MutCompute	(4)
2021	Bst DNA polymerase	stability	self-supervised 3D convolutional neural network MutCompute	(5)
2022	de novo proteins	soluble expression	self-supervised graph neural network ProteinMPNN	(6)
2022	dehalogenase	activity	EnzymeMiner with the gradient boosting machine-based predictor SoluProt	(7)

2022	fluorinase	activity	EnzymeMiner with the gradient boosting machine-based predictor SoluProt	(8)
2023	luciferase	activity	self-supervised graph neural network ProteinMPNN	(9)
2023	de novo proteins	various	diffusion model RFdiffusion	(10)
2021	ancestral luciferase	dual activity	PLS regression	(11)
2023	antibodies	protein binding	Bayesian optimization	(12)
2023	amine transaminase	activity	ML-guided directed evolution	(13)
2023	3-(3-hydroxy-decanoyloxy)decanoate synthase	specificity	Bayesian optimization	(14)
2023	luciferase	activity	reinforcement learning framework EvoPlay	(15)
2022	NADP+-dependent malic enzyme	cofactor specificity	logistic regression	(16)
2022	Cas9 protein	editing activity	ML-guided directed evolution	(17)
2022	iron/α-ketoglutarate dependent halogenase	activity, selectivity	Gaussian processes	(18)
2022	antibodies	protein binding	logistic regression	(19)
2022	multiple proteins	unnatural amino acid incorporation	logistic regression	(20)
2021	transpeptidase	activity	ML-guided directed evolution	(21)
2021	alcohol-forming fatty acyl reductases	activity	ML-guided directed evolution	(22)
2021	TEM-1 β-lactamase	activity	LSTM neural network ECNet	(23)
2021	lipase	thermostability	LASSO regression	(24)
2021	B1 domain of protein G	protein binding	convolutional neural networks	(25)
2021	GFP, TEM-1 β-lactamase	activity	fine-tuned large language model UniRep	(26)
2021	epoxide hydrolase	stability	PLS-based nethod innov'SAR	(27)
2021	luciferase	activity, solubility	Variational autoencoder	(28)
2021	B1 domain of protein G	protein binding	linear and logistic regressions	(29)
2021	amine transaminase	thermostability	PLS regression	(30)
2020	aldolase	activity	Gaussian processes	(31)
2020	TEM-1 β-lactamase	activity	self-supervised 3D convolutional neural network MutCompute	(32)
2020	aldehyde deformylating oxygenase	activity	logistic regression	(33)
2020	light-gated ion channel	light sensitivity	Gaussian process	(34)

Table S2. The list of databases and datasets for machine learning applications in protein engineering mentioned in the main paper.

Category	Title	Property	Size	Link	Ref
Sequences	BFD	A large collection of protein sequences	2.5 billion	https://bfd.mmseqs.com/	(35)
	Pfam	protein family categories based on their shared structural and functional characteristics	> 19000 entries	http://pfam.xfam.org/	(36)
	UniProt	protein sequences and functional information	248 million sequences	https://www.uniprot.org/	(37)
	UniRef[100,90,50]	Sequences from UniProtKB and UniParc clustered at 100%, 90%, or 50% sequence identity	356, 170, 60 million sequences in UniRef[100,90,50]	http://www.uniprot.org/uniref	(38)
Structures	PDB	experimentally determined protein structures	>20000 structures	https://www.rcsb.org/	(39)
	AlphaFoldDB	predicted protein structures	>200 million structures from UniProt sequences	https://alphafold.ebi.ac.uk/	(40)
	CATH	protein structure and classification based on their structural features and evolutionary relations	>150 million protein domains, classified to more than 5500 superfamilies	https://www.cathdb.info/	(41)
	SUPERFAMILY	structural and functional annotation for all proteins and genomes	>3000 distinct organisms	https://supfam.mrc-lmb.cam.ac.uk/SUPERFAMILY/	(42)
	PDB-redo	enhanced versions of pre-existing entries in the PDB	~117000 structures	https://pdb-redo.eu/	(43)
Function	BRENDA	enzyme functional data	>8000 different enzymes	https://www.brenda-enzymes.org/	(44)
	SABIO-RK	curated biochemical reaction kinetics	>73000 curated entries	http://sabio.h-its.org/	(45)
	PathBank	pathways of model organisms	100000 machine-readable pathways	https://pathbank.org/	(46)
	ATLAS	biochemical reactions	>149000 known and novel reactions	https://lcsb-databases.epfl.ch/pathways/atlas/	(47)
	MetaNetX	genome-scale metabolic networks and biochemical pathways	>37000 reactions	https://www.metanetx.org/	(48)
	ENZYME database	Enzyme Commission (EC) numbers	>6000 active entries	https://enzyme.expasy.org/	(49)

	ECREACT	postprocessed enzymatic reactions	>62000 unique reaction–EC number combinations	https://github.com/rxn4chemistry/biocatalysis-model	(50)
	EnzymeMap	postprocessed enzymatic reactions	>132000 reactions	https://github.com/hesther/enzymemap	(51)
	Rhea-a	expert-curated biochemical reactions	15876 reactions	http://www.ebi.ac.uk/rhea	(52)
	D3DistalMutation	relation of distal mutations to enzyme activity	7201 proteins	https://www.d3pharma.com/D3DistalMutation/index.php	(53)
Solubility	TargetTrack	massive crystallisation experiments	350000 protein sequences	https://zenodo.org/record/821654	(54)
	SoluProtMutDB	protein solubility data pre-processed for machine learning	32992 sequences	https://loschmidt.chemi.muni.cz/soluprotmutdb/	(55)
Stability	Meltome Atlas	protein stability data across 13 organisms from mass-spectrometry	48000 proteins	https://meltomeatlas.proteomics.wzw.tum.de/master_meltomeatlasapp/	(56)
	FireProtDB	protein stability data for single-point mutants	15987 entries	https://loschmidt.chemi.muni.cz/fireprotdb/	(57)
	ThermoMutDB	mutational data for protein stability	>14669 entries	https://biosig.lab.uq.edu.au/thermomutdb/	(58)
	ProThermDB	thermodynamic data for wild-type and mutants proteins	31500 entries	https://web.iitm.ac.in/bioinfo2/prothermdb/index.html	(59)
Binding	SKEMPI 2.0	binding free energy changes upon mutation for PPI	7085 mutations	https://life.bsc.es/pid/skempi2/	(60)
Aggregation	WALTZ-DB	amyloid aggregation prone sequences	512 amyloid sequences	http://waltzdb.switchlab.org/	(61)
	CPAD 2.0	mechanistic and kinetic aspects of protein aggregation	various aggregation related annotations, 565 annotated structures and around 83000 kinetic entries	https://web.iitm.ac.in/bioinfo2/cpad2/index.html	(62)
Benchmarks	VariBench	various benchmark datasets for variation effect prediction	419 datasets, 329 million variants	http://structure.bmc.lu.se/VariBench/	(63)
	TAPE	biologically relevant semi-supervised learning tasks	5 datasets of various sizes from 8 up to 53 thousand entries	https://github.com/songlab-cal/tape	(64)
	FLIP	datasets for function prediction	3 mutational datasets from 8 up to 290000 of sequences	https://benchmark.protein.properties/	(65)

	PROBE	benchmarks for measuring protein representation model performance	4 benchmark datasets of various sizes from 20000 to 50 million entries	https://github.com/kansil/PROBE	(66)
	ProteinGym	a set of deep mutational scanning for protein fitness, including the effects of InDels	1.5 million missense mutant variants and 300000 InDel mutants	https://www.proteingym.org/	(67)

References

1. Russ, W.P., Figliuzzi, M., Stocker, C., Barrat-Charlaix, P., Socolich, M., Kast, P., Hilvert, D., Monasson, R., Cocco, S., Weigt, M. and Ranganathan, R., 2020. An evolution-based model for designing chorismate mutase enzymes. Science, 369(6502), pp.440-445.
2. Repecka, D., Jauniskis, V., Karpus, L., Rembeza, E., Rokaitis, I., Zrimec, J., Poviloniene, S., Laurynenas, A., Viknander, S., Abuajwa, W. and Savolainen, O., 2021. Expanding functional protein sequence spaces using generative adversarial networks. Nature Machine Intelligence, 3(4), pp.324-333.
3. Madani, A., Krause, B., Greene, E.R., Subramanian, S., Mohr, B.P., Holton, J.M., Olmos Jr, J.L., Xiong, C., Sun, Z.Z., Socher, R. and Fraser, J.S., 2023. Large language models generate functional protein sequences across diverse families. Nature Biotechnology, pp.1-8.
4. Lu, H., Diaz, D.J., Czarnecki, N.J., Zhu, C., Kim, W., Shroff, R., Acosta, D.J., Alexander, B.R., Cole, H.O., Zhang, Y. and Lynd, N.A., 2022. Machine learning-aided engineering of hydrolases for PET depolymerization. Nature, 604(7907), pp.662-667.
5. Paik, I., Ngo, P.H., Shroff, R., Diaz, D.J., Maranhao, A.C., Walker, D.J., Bhadra, S. and Ellington, A.D., 2021. Improved bst DNA polymerase variants derived via a machine learning approach. Biochemistry, 62(2), pp.410-418.
6. Dauparas, J., Anishchenko, I., Bennett, N., Bai, H., Ragotte, R.J., Milles, L.F., Wicky, B.I., Courbet, A., de Haas, R.J., Bethel, N. and Leung, P.J., 2022. Robust deep learning–based protein sequence design using ProteinMPNN. Science, 378(6615), pp.49-56.
7. Vasina, M., Vanacek, P., Hon, J., Kovar, D., Faldynova, H., Kunka, A., Buryska, T., Badenhorst, C.P., Mazurenko, S., Bednar, D. and Stavrakis, S., 2022. Advanced database mining of efficient haloalkane dehalogenases by sequence and structure bioinformatics and microfluidics. Chem Catalysis, 2(10), pp.2704-2725.
8. Pardo, I., Bednar, D., Calero, P., Volke, D.C., Damborský, J. and Nikel, P.I., 2022. A nonconventional archaeal fluorinase identified by in silico mining for enhanced fluorine biocatalysis. ACS catalysis, 12(11), pp.6570-6577.
9. Yeh AH, Norn C, Kipnis Y, Tischer D, Pellock SJ, Evans D, Ma P, Lee GR, Zhang JZ, Anishchenko I, Coventry B. De novo design of luciferases using deep learning. Nature. 2023 Feb 23;614(7949):774-80.
10. Watson, J.L., Juergens, D., Bennett, N.R., Trippe, B.L., Yim, J., Eisenach, H.E., Ahern, W., Borst, A.J., Ragotte, R.J., Milles, L.F. and Wicky, B.I., 2023. De novo design of protein structure and function with RFdiffusion. Nature, pp.1-3.
11. Schenkmayerova, A., Pinto, G.P., Toul, M., Marek, M., Hernychova, L., Planas-Iglesias, J., Daniel Liskova, V., Pluskal, D., Vasina, M., Emond, S. and Dörr, M., 2021. Engineering the protein dynamics of an ancestral luciferase. Nature Communications, 12(1), p.3616.
12. Li, L., Gupta, E., Spaeth, J., Shing, L., Jaimes, R., Engelhart, E., Lopez, R., Caceres, R.S., Bepler, T. and Walsh, M.E., 2023. Machine learning optimization of candidate antibody yields highly diverse sub-nanomolar affinity antibody libraries. Nature Communications, 14(1), p.3454.
13. Ao, Y.F., Pei, S., Xiang, C., Menke, M.J., Shen, L., Sun, C., Dörr, M., Born, S., Höhne, M. and Bornscheuer, U.T., 2023. Struktur-und Daten-basiertes Protein Engineering von Transaminasen zur Verbesserung von Aktivität und Stereoselektivität. Angewandte Chemie, 135(23), p.e202301660.
14. Hu, R., Fu, L., Chen, Y., Chen, J., Qiao, Y. and Si, T., 2023. Protein engineering via Bayesian optimization-guided evolutionary algorithm and robotic experiments. Briefings in Bioinformatics, 24(1), p.bbac570.
15. Wang, Y., Tang, H., Huang, L., Pan, L., Yang, L., Yang, H., Mu, F. and Yang, M., 2023. Self-play reinforcement learning guides protein engineering. Nature Machine Intelligence, pp.1-16.
16. Sugiki, S., Niide, T., Toya, Y. and Shimizu, H., 2022. Logistic Regression-Guided Identification of Cofactor Specificity-Contributing Residues in Enzyme with Sequence Datasets Partitioned by Catalytic Properties. ACS Synthetic Biology, 11(12), pp.3973-3985.
17. Thean, D.G., Chu, H.Y., Fong, J.H., Chan, B.K., Zhou, P., Kwok, C.C., Chan, Y.M., Mak, S.Y., Choi, G.C., Ho, J.W. and Zheng, Z., 2022. Machine learning-coupled combinatorial mutagenesis enables resource-efficient engineering of CRISPR-Cas9 genome editor activities. Nature Communications, 13(1), p.2219.
18. Büchler, J., Malca, S.H., Patsch, D., Voss, M., Turner, N.J., Bornscheuer, U.T., Allemann, O., Le Chapelain, C., Lumbroso, A., Loiseleur, O. and Buller, R., 2022. Algorithm-aided engineering of aliphatic halogenase WelO5* for the asymmetric late-stage functionalization of soraphens. Nature Communications, 13(1), p.371.
19. Makowski, E.K., Chen, H., Lambert, M., Bennett, E.M., Eschmann, N.S., Zhang, Y., Zupancic, J.M., Desai, A.A., Smith, M.D., Lou, W. and Fernando, A., 2022, December. Reduction of therapeutic antibody self-association using yeast-display selections and machine learning. In MAbs (Vol. 14, No. 1, p. 2146629). Taylor & Francis.
20. Zhang, H., Zheng, Z., Dong, L., Shi, N., Yang, Y., Chen, H., Shen, Y. and Xia, Q., 2022. Rational incorporation of any unnatural amino acid into proteins by machine learning on existing experimental proofs. Computational and Structural Biotechnology Journal, 20, pp.4930-4941.
21. Saito, Y., Oikawa, M., Sato, T., Nakazawa, H., Ito, T., Kameda, T., Tsuda, K. and Umetsu, M., 2021. Machine-learning-guided library design cycle for directed evolution of enzymes: the effects of training data composition on sequence space exploration. ACS Catalysis, 11(23), pp.14615-14624.
22. Greenhalgh, J.C., Fahlberg, S.A., Pfleger, B.F. and Romero, P.A., 2021. Machine learning-guided acyl-ACP reductase engineering for improved in vivo fatty alcohol production. Nature communications, 12(1), p.5825.
23. Luo, Y., Jiang, G., Yu, T., Liu, Y., Vo, L., Ding, H., Su, Y., Qian, W.W., Zhao, H. and Peng, J., 2021. ECNet is an evolutionary context-integrated deep learning framework for protein engineering. Nature communications, 12(1), p.5743.
24. Yoshida, K., Kawai, S., Fujitani, M., Koikeda, S., Kato, R. and Ema, T., 2021. Enhancement of protein thermostability by three consecutive mutations using loop-walking method and machine learning. scientific reports, 11(1), p.11883.
25. Gelman, S., Fahlberg, S.A., Heinzelman, P., Romero, P.A. and Gitter, A., 2021. Neural networks to learn protein sequence–function relationships from deep mutational scanning data. Proceedings of the National Academy of Sciences, 118(48), p.e2104878118.
26. Biswas, S., Khimulya, G., Alley, E.C., Esvelt, K.M. and Church, G.M., 2021. Low-N protein engineering with data-efficient deep learning. Nature methods, 18(4), pp.389-396.
27. Li, G., Qin, Y., Fontaine, N.T., Ng Fuk Chong, M., Maria-Solano, M.A., Feixas, F., Cadet, X.F., Pandjaitan, R., Garcia-Borràs, M., Cadet, F. and Reetz, M.T., 2021. Machine learning enables selection of epistatic enzyme mutants for stability against unfolding and detrimental aggregation. ChemBioChem, 22(5), pp.904-914.
28. Hawkins-Hooker, A., Depardieu, F., Baur, S., Couairon, G., Chen, A. and Bikard, D., 2021. Generating functional protein variants with variational autoencoders. PLoS computational biology, 17(2), p.e1008736.
29. Song, H., Bremer, B.J., Hinds, E.C., Raskutti, G. and Romero, P.A., 2021. Inferring protein sequence-function relationships with large-scale positive-unlabeled learning. Cell systems, 12(1), pp.92-101.
30. Jia, L.L., Sun, T.T., Wang, Y. and Shen, Y., 2021. A machine learning study on the thermostability prediction of (R)-ω-selective amine transaminase from Aspergillus terreus. BioMed Research International, 2021.
31. Voutilainen, S., Heinonen, M., Andberg, M., Jokinen, E., Maaheimo, H., Pääkkönen, J., Hakulinen, N., Rouvinen, J., Lähdesmäki, H., Kaski, S. and Rousu, J., 2020. Substrate specificity of 2-deoxy-D-ribose 5-phosphate aldolase (DERA) assessed by different protein engineering and machine learning methods. Applied Microbiology and Biotechnology, 104, pp.10515-10529.
32. Shroff, R., Cole, A.W., Diaz, D.J., Morrow, B.R., Donnell, I., Annapareddy, A., Gollihar, J., Ellington, A.D. and Thyer, R., 2020. Discovery of novel gain-of-function mutations guided by structure-based deep learning. ACS synthetic biology, 9(11), pp.2927-2935.
33. Mak, W.S., Wang, X., Arenas, R., Cui, Y., Bertolani, S., Deng, W.Q., Tagkopoulos, I., Wilson, D.K. and Siegel, J.B., 2020. Discovery, design, and structural characterization of alkane-producing enzymes across the ferritin-like superfamily. Biochemistry, 59(40), pp.3834-3843.

34. Ikegami, K., de March, C.A., Nagai, M.H., Ghosh, S., Do, M., Sharma, R., Bruguera, E.S., Lu, Y.E., Fukutani, Y., Vaidehi, N. and Yohda, M., 2020. Structural instability and divergence from conserved residues underlie intracellular retention of mammalian odorant receptors. Proceedings of the National Academy of Sciences, 117(6), pp.2957-2967.
35. Jumper, J.; Evans, R.; Pritzel, A.; Green, T.; Figurnov, M.; Ronneberger, O.; Tunyasuvunakool, K.; Bates, R.; Žídek, A.; Potapenko, A.; Bridgland, A.; Meyer, C.; Kohl, S. A. A.; Ballard, A. J.; Cowie, A.; Romera-Paredes, B.; Nikolov, S.; Jain, R.; Adler, J.; Back, T.; Petersen, S.; Reiman, D.; Clancy, E.; Zielinski, M.; Steinegger, M.; Pacholska, M.; Berghammer, T.; Bodenstein, S.; Silver, D.; Vinyals, O.; Senior, A. W.; Kavukcuoglu, K.; Kohli, P.; Hassabis, D. Highly Accurate Protein Structure Prediction with AlphaFold. Nature 2021, 596 (7873), 583–589.
36. Mistry, J., Chuguransky, S., Williams, L., Qureshi, M., Salazar, G.A., Sonnhammer, E.L.L., Tosatto, S.C.E., Paladin, L., Raj, S., Richardson, L.J., Finn, R.D., Bateman, A., 2021. Pfam: The protein families database in 2021. Nucleic Acids Res. 49, D412–D419. https://doi.org/10.1093/nar/gkaa913
37. UniProt: the universal protein knowledgebase in 2023. Nucleic Acids Research, 2023, 51.D1: D523-D531.
38. Suzek, B. E.; Wang, Y.; Huang, H.; McGarvey, P. B.; Wu, C. H.; UniProt Consortium. UniRef Clusters: A Comprehensive and Scalable Alternative for Improving Sequence Similarity Searches. Bioinformatics 2015, 31 (6), 926–932.
39. Burley, S.K., Bhikadiya, C., Bi, C., Bittrich, S., Chao, H., Chen, L., Craig, P.A., Crichlow, G.V., Dalenberg, K., Duarte, J.M., Dutta, S., Fayazi, M., Feng, Z., Flatt, J.W., Ganesan, S.J., Ghosh, S., Goodsell, D.S., Green, R.K., Guranovic, V., Henry, J., Hudson, B.P., Khokhriakov, I., Lawson, C.L., Liang, Y., Lowe, R., Peisach, E., Persikova, I., Piehl, D.W., Rose, Y., Sali, A., Segura, J., Sekharan, M., Shao, C., Vallat, B., Voigt, M., Webb, B., Westbrook, J.D., Whetstone, S., Young, J.Y., Zalevsky, A., Zardecki, C., 2022. RCSB Protein Data bank: Tools for visualizing and understanding biological macromolecules in 3D. Protein Sci. 31, e4482.
40. Varadi, M.; Anyango, S.; Deshpande, M.; Nair, S.; Natassia, C.; Yordanova, G.; Yuan, D.; Stroe, O.; Wood, G.; Laydon, A.; Žídek, A.; Green, T.; Tunyasuvunakool, K.; Petersen, S.; Jumper, J.; Clancy, E.; Green, R.; Vora, A.; Lutfi, M.; Figurnov, M.; Cowie, A.; Hobbs, N.; Kohli, P.; Kleywegt, G.; Birney, E.; Hassabis, D.; Velankar, S. AlphaFold Protein Structure Database: Massively Expanding the Structural Coverage of Protein-Sequence Space with High-Accuracy Models. Nucleic Acids Res. 2022, 50 (D1), D439–D444.
41. Sillitoe, I.; Bordin, N.; Dawson, N.; Waman, V. P.; Ashford, P.; Scholes, H. M.; Pang, C. S. M.; Woodridge, L.; Rauer, C.; Sen, N.; Abbasian, M.; Le Cornu, S.; Lam, S. D.; Berka, K.; Varekova, I. H.; Svobodova, R.; Lees, J.; Orengo, C. A. CATH: Increased Structural Coverage of Functional Space. Nucleic Acids Res. 2021, 49 (D1), D266–D273.
42. Pandurangan, A.P., Stahlhacke, J., Oates, M.E., Smithers, B., Gough, J., 2019. The SUPERFAMILY 2.0 database: a significant proteome update and a new webserver. Nucleic Acids Res. 47, D490–D494.
43. van Beusekom B, Touw WG, Tatineni M, Somani S, Rajagopal G, Luo J, Gilliland GL, Perrakis A, Joosten RP. Homology-based hydrogen bond information improves crystallographic structures in the PDB. Protein Sci. 2018 Mar;27(3):798-808. doi: 10.1002/pro.3353. Epub 2017 Dec 8. PMID: 29168245; PMCID: PMC5818736
44. Chang, A., Jeske, L., Ulbrich, S., Hofmann, J., Koblitz, J., Schomburg, I., Neumann-Schaal, M., Jahn, D., Schomburg, D., 2021. BRENDA, the ELIXIR core data resource in 2021: new developments and updates. Nucleic Acids Res. 49, D498–D508.
45. Wittig, U., Rey, M., Weidemann, A., Kania, R., Müller, W., 2018. SABIO-RK: an updated resource for manually curated biochemical reaction kinetics. Nucleic Acids Res. 46, D656–D660.
46. Wishart, D.S., Li, C., Marcu, A., Badran, H., Pon, A., Budinski, Z., Patron, J., Lipton, D., Cao, X., Oler, E., Li, K., Paccoud, M., Hong, C., Guo, A.C., Chan, C., Wei, W., Ramirez-Gaona, M., 2020. PathBank: a comprehensive pathway database for model organisms. Nucleic Acids Res. 48, D470–D478.
47. Hafner, J., MohammadiPeyhani, H., Sveshnikova, A., Scheidegger, A., Hatzimanikatis, V., 2020. Updated ATLAS of Biochemistry with New Metabolites and Improved Enzyme Prediction Power. ACS Synth. Biol. 9, 1479–1482.
48. Moretti, S., Tran, V.D.T., Mehl, F., Ibberson, M., Pagni, M., 2021. MetaNetX/MNXref: unified namespace for metabolites and biochemical reactions in the context of metabolic models. Nucleic Acids Res. 49, D570–D574.
49. Bairoch, A., 2000. The ENZYME database in 2000. Nucleic Acids Res. 28, 304–305.
50. Probst, D., Manica, M., Nana Teukam, Y.G., Castrogiovanni, A., Paratore, F., Laino, T., 2022. Biocatalysed synthesis planning using data-driven learning. Nat. Commun. 13, 964.
51. Heid, E., Probst, D., Green, W.H., Madsen, G.K.H., 2023. EnzymeMap: Curation, validation and data-driven prediction of enzymatic reactions.
52. Alcántara, R.; Axelsen, K. B.; Morgat, A.; Belda, E.; Coudert, E.; Bridge, A.; Cao, H.; de Matos, P.; Ennis, M.; Turner, S.; Owen, G.; Bougueleret, L.; Xenarios, I.; Steinbeck, C. Rhea--a Manually Curated Resource of Biochemical Reactions. Nucleic Acids Res. 2012, 40 (Database issue), D754–D760.
53. Wang, X., Zhang, X., Peng, C., Shi, Y., Li, H., Xu, Z. and Zhu, W., 2021. D3distalmutation: a database to explore the effect of distal mutations on enzyme activity. Journal of Chemical Information and Modeling, 61(5), pp.2499-2508.
54. Helen M. Berman, M.J.G., Andrei Kouranov, David I. Micallef, John Westbrook, investigators, P.S.I. network of, 2017. Protein Structure Initiative - TargetTrack 2000-2017 - all data files.
55. Velecký, J., Hamsikova, M., Stourac, J., Musil, M., Damborsky, J., Bednar, D. and Mazurenko, S., 2022. SoluProtMutDB: A manually curated database of protein solubility changes upon mutations. Computational and Structural Biotechnology Journal, 20, pp.6339-6347.
56. Jarzab, A., Kurzawa, N., Hopf, T., Moerch, M., Zecha, J., Leijten, N., Bian, Y., Musiol, E., Maschberger, M., Stoehr, G., Becher, I., Daly, C., Samaras, P., Mergner, J., Spanier, B., Angelov, A., Werner, T., Bantscheff, M., Wilhelm, M., Klingenspor, M., Lemeer, S., Liebl, W., Hahne, H., Savitski, M.M., Kuster, B., 2020. Meltome atlas—thermal proteome stability across the tree of life. Nat. Methods 17, 495–503.
57. Stourac, J., Dubrava, J., Musil, M., Horackova, J., Damborsky, J., Mazurenko, S. and Bednar, D., 2021. FireProtDB: database of manually curated protein stability data. Nucleic acids research, 49(D1), pp.D319-D324.
58. Xavier, J.S., Nguyen, T.B., Karmarkar, M., Portelli, S., Rezende, P.M., Velloso, J.P., Ascher, D.B. and Pires, D.E., 2021. ThermoMutDB: a thermodynamic database for missense mutations. Nucleic acids research, 49(D1), pp.D475-D479.
59. Nikam, R., Kulandaisamy, A., Harini, K., Sharma, D. and Gromiha, M.M., 2021. ProThermDB: thermodynamic database for proteins and mutants revisited after 15 years. Nucleic acids research, 49(D1), pp.D420-D424.
60. Jankauskaitė, J., Jiménez-García, B., Dapkūnas, J., Fernández-Recio, J. and Moal, I.H., 2019. SKEMPI 2.0: an updated benchmark of changes in protein–protein binding energy, kinetics and thermodynamics upon mutation. Bioinformatics, 35(3), pp.462-469.
61. Louros, N., Konstantoulea, K., De Vleeschouwer, M., Ramakers, M., Schymkowitz, J. and Rousseau, F., 2020. WALTZ-DB 2.0: an updated database containing structural information of experimentally determined amyloid-forming peptides. Nucleic acids research, 48(D1), pp.D389-D393.
62. Rawat, P., Prabakaran, R., Sakthivel, R., Mary Thangakani, A., Kumar, S. and Gromiha, M.M., 2020. CPAD 2.0: a repository of curated experimental data on aggregating proteins and peptides. Amyloid, 27(2), pp.128-133.
63. Sarkar, A., Yang, Y. and Vihinen, M., 2020. Variation benchmark datasets: update, criteria, quality and applications. Database, 2020, p.baz117.
64. Rao, R., Bhattacharya, N., Thomas, N., Duan, Y., Chen, P., Canny, J., Abbeel, P. and Song, Y., 2019. Evaluating protein transfer learning with TAPE. Advances in neural information processing systems, 32.
65. Dallago, C., Mou, J., Johnston, K.E., Wittmann, B.J., Bhattacharya, N., Goldman, S., Madani, A. and Yang, K.K., 2021. FLIP: Benchmark tasks in fitness landscape inference for proteins. bioRxiv, pp.2021-11.
66. Unsal, S., Atas, H., Albayrak, M., Turhan, K., Acar, A.C. and Doğan, T., 2022. Learning functional properties of proteins with language models. Nature Machine Intelligence, 4(3), pp.227-245.
67. Notin, P., Dias, M., Frazer, J., Hurtado, J.M., Gomez, A.N., Marks, D. and Gal, Y., 2022, June. Tranception: protein fitness prediction with autoregressive transformers and inference-time retrieval. In International Conference on Machine Learning (pp. 16990-17017). PMLR.
